# RNA-binding proteins La and HuR cooperatively modulate translation repression of *PDCD4* mRNA

**DOI:** 10.1074/jbc.RA120.014894

**Published:** 2020-12-09

**Authors:** Ravi Kumar, Dipak Kumar Poria, Partho Sarothi Ray

**Affiliations:** Department of Biological Sciences, Indian Institute of Science Education and Research, Kolkata, Mohanpur, Nadia, West Bengal, India

**Keywords:** ELAV-like protein 1 (HuR [human antigen R]), lupus antigen (La), translation regulation, miRNA, lipopolysaccharide (LPS), programmed cell death 4 (PDCD4), HuR, human antigen R, La, lupus antigen, LPS, lipopolysaccharide, PDCD4, programmed cell death 4, RBPs, RNA-binding proteins, 3ʹUTR, 3ʹ-untranslated region

## Abstract

Posttranscriptional regulation of gene expression plays a critical role in controlling the inflammatory response. An uncontrolled inflammatory response results in chronic inflammation, often leading to tumorigenesis. Programmed cell death 4 (PDCD4) is a proinflammatory tumor-suppressor gene which helps to prevent the transition from chronic inflammation to cancer. PDCD4 mRNA translation is regulated by an interplay between the oncogenic microRNA miR-21 and the RNA-binding protein (RBP) human antigen R (HuR) in response to lipopolysaccharide stimulation, but the role of other regulatory factors remains unknown. Here, we report that the RBP lupus antigen (La) interacts with the 3ʹ-untranslated region of PDCD4 mRNA and prevents miR-21-mediated translation repression. While lipopolysaccharide causes nuclear-cytoplasmic translocation of HuR, it enhances cellular La expression. Remarkably, La and HuR were found to bind cooperatively to the PDCD4 mRNA and mitigate miR-21-mediated translation repression. The cooperative action of La and HuR reduced cell proliferation and enhanced apoptosis, reversing the pro-oncogenic function of miR-21. Together, these observations demonstrate a cooperative interplay between two RBPs, triggered differentially by the same stimulus, which exerts a synergistic effect on PDCD4 expression and thereby helps maintain a balance between inflammation and tumorigenesis.

Posttranscriptional regulation of proinflammatory gene expression plays an important role in controlling the inflammatory response, particularly at the stage of inflammation resolution (1). Resolution of inflammation is an integrated process that restores tissue homeostasis once the inflammatory insult has been mitigated (2, 3). Failure to resolve inflammation leads to chronic inflammation, marked by the persistent expression of proinflammatory mediators and consequent tissue damage, and can lead to multiple disease conditions including cancer (4, 5).

Posttranscriptional processes that control the translation/turnover of mRNAs of proinflammatory genes play crucial roles in regulating the expression of inflammatory mediators (6, 7, 8, 9). Translation regulation is mediated by the signal-induced binding of RNA-binding proteins (RBPs) or noncoding RNAs such as long noncoding RNAs and miRNAs to specific sequences or structural elements in the 5ʹ- and 3ʹ-untranslated regions (UTRs) of mRNAs (10, 11, 12, 13). About 692 RBPs have been discovered till date which interact with mRNAs in both cytoplasm and nucleus (14). Coordinated assembly of these RBPs on target mRNAs regulates mRNA translation or stability, often *via* cooperative or competitive interactions (15, 16, 17, 18). External stimuli rapidly modulate RNA binding activity of RBPs through changes in expression levels, nucleocytoplasmic translocation, or posttranslational modifications (19). Increasingly, RBPs have also been found to fine-tune gene regulation by crosstalk with miRNAs, either by collaborative or competitive interplay between RBP and miRNA binding to target mRNAs (20, 21, 22, 23).

Programmed cell death 4 (*PDCD4*) is a proinflammatory tumor suppressor gene which regulates apoptosis of cells in response to inflammatory stimuli. PDCD4 acts as a tumor suppressor by inhibiting malignant transformation, tumor progression, and metastasis (24). PDCD4 is induced by inflammatory stimuli and functions by activating the proinflammatory transcription factor nuclear factor kappa-B (NFκB) and inhibiting the ERK/p38 MAPK pathways, thereby suppressing the expression of antiinflammatory cytokine interleukin-10 (IL-10) (25, 26). It is also induced by apoptotic stimuli and causes programmed cell death by repressing translation, most likely by binding to the translation initiation factor eIF4A or by interacting with structured 5ʹ-UTRs of specific mRNAs (27, 28, 29). PDCD4 also acts as a negative regulator of the cell cycle by inhibiting AP1-dependent transcription (30). Therefore, PDCD4 plays an important multifunctional role in maintaining the balance between inflammation and tumorigenesis.

PDCD4 expression is negatively regulated by the “oncomiR” miR-21. miR-21 directly interacts with the *PDCD4* mRNA *via* a specific target site (nt 228–249) within the 3ʹ-UTR and represses its translation (31, 32, 33). Increased expression of miR-21 has been implicated in various processes involved in carcinogenesis, including inhibition of apoptosis, promotion of cell proliferation, and stimulation of tumor growth (34, 35). miR-21 also acts as an important regulator of PDCD4 in monocytes in response to stimulation by bacterial lipopolysaccharide (LPS) (36).

The RBP human antigen R (HuR) is reported to bind to the 3ʹ-untranslated region (3ʹUTR) of *PDCD4* mRNA and regulate its translation (37, 38). HuR or ELAVL1 is a ubiquitously expressed RNA-binding protein belonging to the ELAV (Embryonic Lethal Abnormal Vision) family which binds to A- and/or U-rich elements (A/UREs) in 3ʹUTRs of mRNAs (39, 40). Target mRNAs of HuR are involved in various processes such as cell proliferation, apoptosis, angiogenesis, inflammation, and stress response (41). HuR is predominantly found in nucleus but undergoes nuclear-cytoplasmic translocation in response to external stimuli such as UV radiation, inflammatory agonists, hypoxia, nutrient deprivation, and oxidative stress (42, 43, 44, 45, 46). In the cytoplasm, HuR binds to various mRNAs and regulate their translation and/or stability (47, 48). Previous work has shown that HuR binds to the *PDCD4* 3ʹUTR after nuclear-cytoplasmic translocation in response to treatment with the inflammatory agonist LPS (37). HuR binding to the *PDCD4* 3ʹUTR prevents binding of the miR-21–RISC complex to the mRNA. Moreover, HuR was also found to act as a “microRNA sponge” by directly binding to miR-21 and sequestering it (37). This dual role of HuR prevented miR-21–mediated translation repression of *PDCD4* RNA in response to inflammatory stimulus. Besides HuR, the only other RBP that has been reported to bind to the PDCD4 mRNA 3ʹUTR is the T-cell-restricted intracellular antigen 1 (TIA-1) which repressed PDCD4 expression (38).

In this study, we have taken an unbiased approach to identify RBPs binding to the *PDCD4* mRNA 3ʹUTR and have identified lupus antigen (La) as one of the interacting partners of the *PDCD4* mRNA 3ʹUTRs. The inflammatory agonist LPS, which has been shown to cause nuclear-cytoplasmic shuttling of HuR, causes induction of La expression. Interestingly, La protein cooperates with HuR in binding to the 3ʹUTR and reversing the miR-21–mediated repression of *PDCD4* mRNA translation, thereby causing a synergistic effect in the induction of PDCD4 expression in response to inflammatory stimulus.

## Results

### Identification of La protein as an interacting partner of the PDCD4 mRNA 3ʹUTR

**Figure 1:**
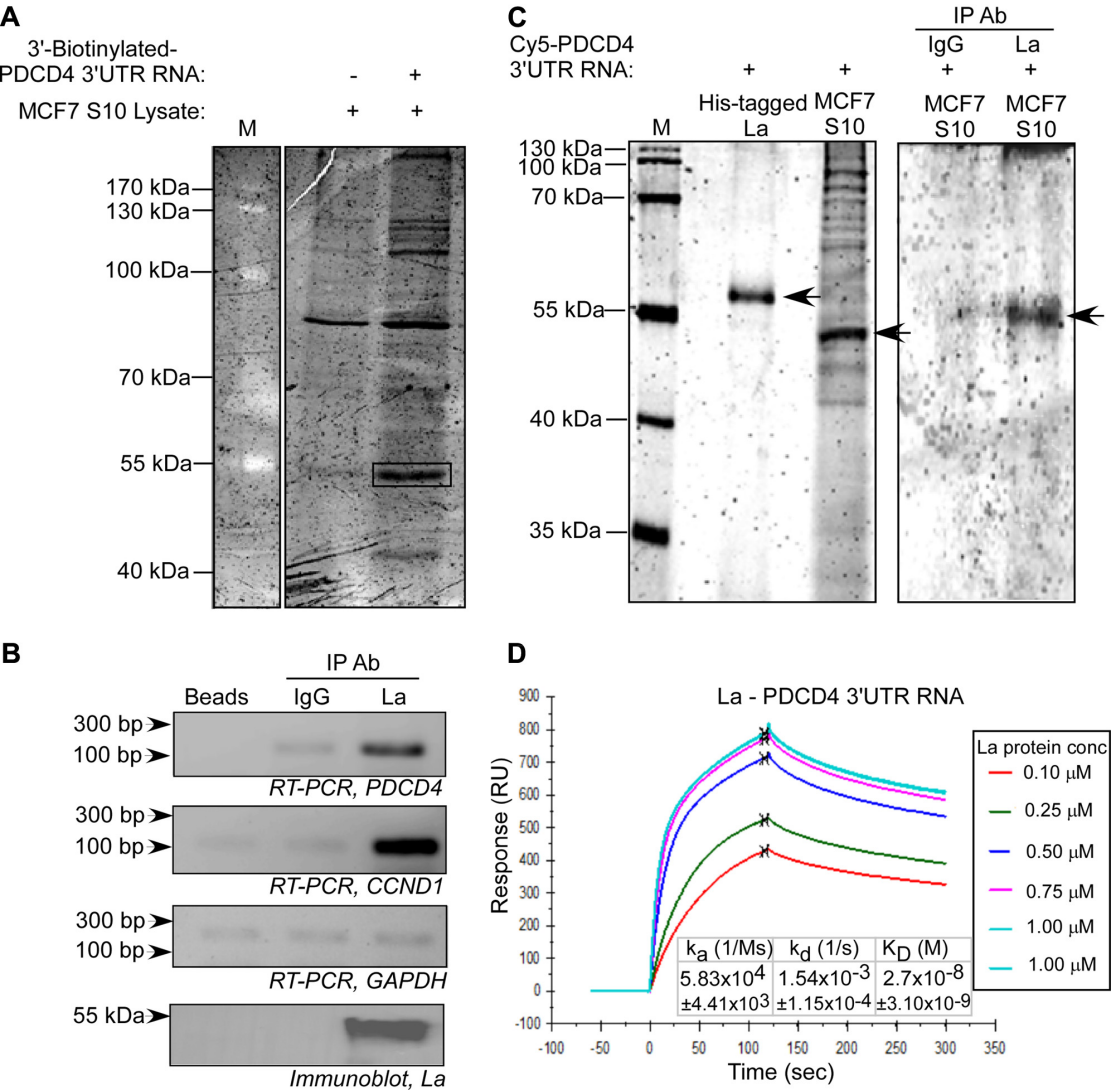
**RNA-binding protein La interacts with *PDCD4* mRNA 3'UTR**. *A*, five hundred micrograms MCF7 cytoplasmic lysate was incubated with 10 μg 3ʹ-biotinylated *PDCD4* 3ʹUTR RNA. Proteins associated with the RNA were purified by affinity chromatography and resolved by 10% SDS-PAGE. Protein band marked by *black rectangle* was found to contain La protein by mass spectrometry. Lane M contains protein molecular weight markers, second lane contains proteins eluted from streptavidin beads only, third lane contains proteins eluted from 3ʹ-biotinylated *PDCD4* 3ʹUTR RNA. *B*, cell lysates were immunoprecipitated with anti-La antibody or control IgG, and immunoprecipitated RNA was isolated and subjected to RT–PCR using *PDCD4*, CyclinD1 (*CCND1*), and *GAPDH* primers. RNA pulled down only with protein-A sepharose beads was analyzed for nonspecific interaction. The panel at the bottom represents immunoblot of La protein from the immunoprecipitate. *C*, Cy5-UTP-labeled *PDCD4* 3ʹUTR RNA was incubated either with purified, recombinant His-tagged La protein or MCF7 cytoplasmic lysate followed by UV crosslinking and RNase A digestion. The RNP complexes were resolved by 10% SDS-PAGE (left panel). The RNP complexes were immunoprecipitated with La antibody or IgG and resolved on 10% SDS-PAGE (right panel). The La RNP-complex bands are indicated by arrows. *D*, *in vitro* transcribed 3ʹ-biotinylated *PDCD4* 3ʹUTR RNA was immobilized on the Biacore SA chip. Increasing concentrations of purified La protein was flowed over the chip, and the Response Units were plotted against time. Binding constants (K_a_, K_d_, and K_D_) were calculated considering 1:1 binding kinetics. The binding constants represent the mean ± SEM from three independent experiments. La, lupus antigen; PDCD4, programmed cell death 4; 3ʹUTR, 3ʹ-untranslated region.

The RBP interactome of the *PDCD4* 3ʹUTR is not well characterized. We adopted an unbiased approach comprising of RNA-affinity chromatography coupled with mass spectrometry to identify RBPs which interact with the 3ʹUTR of *PDCD4* mRNA and potentially regulate *PDCD4* mRNA translation/turnover. 3ʹ-end biotinylated full-length *PDCD4* 3ʹUTR was used to pull down the RNA binding proteins from MCF7 cell lysate, following which bound proteins were resolved by electrophoresis (Fig. 1*A*). LC-MS/MS analysis of a band migrating close to the 55 kDa molecular weight marker yielded human La protein as an RBP component (Table S1). We selected La, lupus antigen, or Sjögren syndrome type B antigen for further investigation as it is a well-known RBP involved in the regulation of translation and replication of multiple viral and cellular mRNAs under various physiological and pathological conditions and in the 3ʹ-end processing and 5ʹ-leader removal of RNA pol III transcripts (49, 50, 51, 52). Moreover, La protein has been previously reported to bind to 5ʹUTRs of multiple mammalian mRNAs but not to 3ʹUTRs. Therefore, the possibility of La binding to the 3ʹUTR of *PDCD4* mRNA motivated us toward further investigation of its role in regulating PDCD4 expression.

The interaction of La protein with *PDCD4* mRNA was verified by immunoprecipitating La protein from MCF7 cell lysate, followed by reverse transcription-PCR, showing specific association of PDCD4 mRNA with the La protein (Fig. 1*B*) Cyclin D1 (*CCND1*) mRNA 5ʹUTR which is known to bind to La protein (53) was taken as positive control. To investigate whether La-*PDCD4* mRNA interaction is mediated through the 3ʹUTR of the mRNA, UV-crosslinking of Cy5-UTP-labeled *PDCD4* 3ʹUTR with purified, His-tagged recombinant La protein and MCF7 cell cytoplasmic lysate demonstrated RNA–protein complexes corresponding to around 57 kDa and 52 kDa in the recombinant protein and MCF lysate, respectively (Fig. 1*C*, lanes 2 and 3). The 52 kDa band was confirmed as the La-*PDCD4* 3ʹUTR complex by immunoprecipitation of the fluorescently labeled *PDCD4* 3ʹUTR RNA–protein complex from MCF7 cytoplasmic lysate using anti-La antibody (Fig. 1*C*). Furthermore, surface plasmon resonance demonstrated the interaction between purified La protein and *PDCD4* mRNA 3ʹUTR (Fig. S1) with an equilibrium dissociation constant (K_D_) of 27 × 10^−9^ M (Fig. 1*D*). As a positive control, we checked the binding of La with miR-125b primary miRNA, as La has been reported to bind to pri-miRNAs and pre-miRNAs with high affinity (54). La showed a high affinity binding with miR-125b pri-miRNA in surface plasmon resonance assay (Fig. S2). Together, these observations suggested that La specifically interacts with the PDCD4 mRNA 3ʹUTR in cells and *in vitro* with high affinity.

### La protein enhances *PDCD4* mRNA translation and reverses miR-21-mediated translation repression

**Figure 2:**
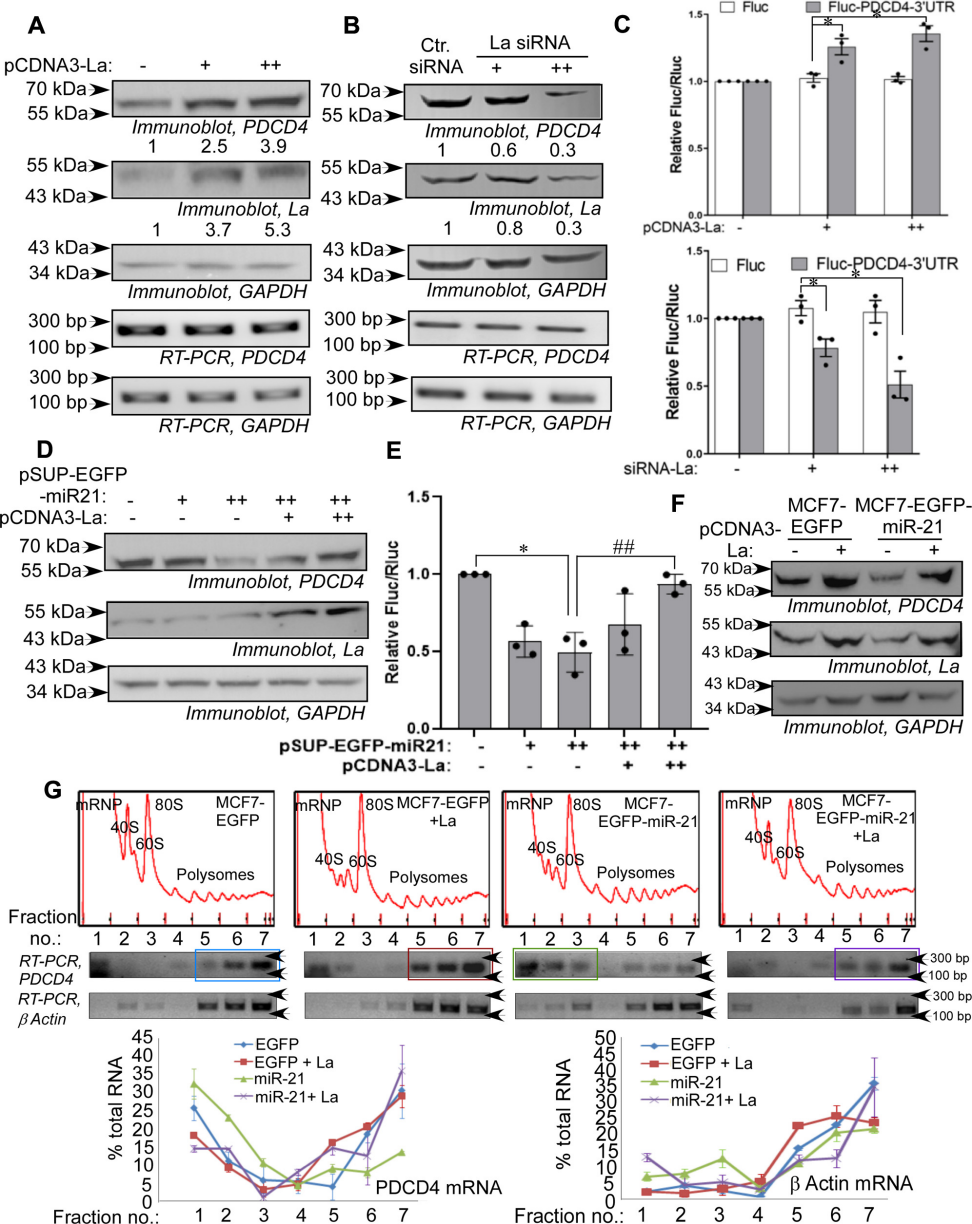
**La protein induces *PDCD4* mRNA translation and reverses miR-21–mediated translation repression**. *A*, immunoblots of MCF7 cell lysates transfected with two increasing concentrations (2 μg and 4 μg) of pCDNA3–La. Immunoblots were probed with anti-PDCD4, anti-La, and anti-GAPDH antibodies. RNA isolated was subjected to RT-PCR using *PDCD4* and *GAPDH* primers (lower two panels). The values indicate the fold change in PDCD4 and La band intensity over control. *B*, immunoblots of MCF7 cell lysates transfected with control siRNA and two increasing concentrations (50 pmole and 100 pmole) of La siRNA. Immunoblots were probed with anti-PDCD4, anti-La, and anti-GAPDH antibodies. RNA isolated from the same cells was subjected to RT-PCR using *PDCD4* and *GAPDH* primers (lower two panels). The values indicate the fold change in PDCD4 and La band intensity over control. *C*, luciferase reporter assay of MCF7 cells co-transfected with Fluc or Fluc-*PDCD4* 3ʹUTR reporter gene constructs and two increasing concentrations of pCDNA3–La (250 ng and 500 ng, upper panel) and La siRNA (5 pmole and 10 pmole, lower panel). Fluc values were normalized to Rluc values as a transfection control. ∗ represents significant difference (*p*-value ≤ 0.05) from respective controls. *D*, immunoblots of lysates of MCF7 cells transfected with two increasing concentrations (1 μg and 2 μg) of pSUPER–miR-21 and cotransfected with two increasing concentrations (1 μg and 2 μg) of pCDNA3-La together with the higher amount of pSUPER–miR-21, probed with anti-PDCD4, anti-La, and anti-GAPDH antibodies. *E*, luciferase reporter assay of MCF7 cells transfected with Fluc-*PDCD4*-3ʹUTR reporter gene construct, cotransfected with two increasing concentrations of pSUPER-miR-21 and of two increasing concentrations (125 ng and 250 ng) of pCDNA3-La in presence of the higher amount of pSUPER-miR-21. Fluc values are normalized to Rluc values as transfection control. ∗Represents significant difference (*p*-value ≤ 0.05) with mock transfected cells and ## represents significant difference (*p*-value ≤ 0.01) with cells transfected with higher dose of pSUP–EGFP–miR21 (++). *F*, lysates from MCF7–EGFP and MCF7–EGFP–miR-21 cell lines transfected or not transfected with pCDNA3-La (2 μg) were immunoblotted with anti-PDCD4, anti-La, and anti-GAPDH antibodies. *G*, ribosomal fractions from MCF7–EGFP and MCF7–EGFP-miR-21 cell lines transfected with pCDNA3-La (5 μg) were analyzed by 10 to 50% sucrose density gradient fractionation. Ribosomal RNA content, measured at 254 nm, was plotted against fraction numbers (upper panel). RNA isolated from selected fractions was analyzed by semi-quantitative RT–PCR using *PDCD4* and *β-actin* primers (middle panels). *Colored boxes* represent position of *PDCD4* mRNA in the fractions. Densitometry value of each band on agarose gel was plotted as a percentage of the sum of density of all bands against fraction number. The data represent mean ± SD from two independent experiments. La, lupus antigen; PDCD4, programmed cell death 4; 3ʹUTR, 3ʹ-untranslated region.

La protein is a regulator of translation of a number of cellular and viral RNAs. Therefore, we checked the effect of La on translation of *PDCD4* mRNA. Overexpression of La protein in MCF7 cells caused an increase in PDCD4 protein level, without altering *PDCD4* mRNA level (Fig. 2*A*), indicating La as a positive regulator of PDCD4 protein synthesis. siRNA-mediated downregulation of La protein strongly reduced PDCD4 expression, without reducing *PDCD4* RNA level (Fig. 2*B*). Furthermore, the effect of La protein on PDCD4 expression was found to be mediated by the 3ʹUTR of *PDCD4* mRNA, as overexpression of La protein showed increased luciferase activity from a *PDCD4* mRNA 3′-UTR containing reporter gene construct (Fig. 2*C*, upper panel). Conversely, siRNA-mediated downregulation of La caused a dose-dependent reduction in reporter gene activity (Fig. 2*C*, lower panel).

The microRNA miR-21 is known to target the *PDCD4* mRNA and repress its translation. Therefore, we investigated the effect of La on miR-21–mediated repression of PDCD4 expression. La prevented the miR-21–mediated inhibition of PDCD4 expression, as expression of increasing concentrations of La protein in MCF7 cells reversed the reduction in PDCD4 protein in presence of miR-21 (Fig. 2*D*). Overexpression of La did not change miR-21 level (Fig. S3). La protein also reversed the miR-21–mediated downregulation of luciferase activity from the Fluc-*PDCD4* 3′-UTR reporter in a dose-dependent manner (Fig. 2*E*). Thereafter, La was overexpressed in an MCF7 cell line stably expressing miR-21 (MCF7–EGFP-miR-21) and a control cell line which expresses only EGFP (MCF7–EGFP) (37). Ectopic overexpression of La was sufficient to rescue the cellular PDCD4 protein level as well as the reporter activity from the FLuc–*PDCD4* 3ʹ-UTR reporter construct when transfected into the miR-21 expressing cell line (Figs. 2*F* and S4).

To check whether La actually reversed the miR-21–mediated translation repression of *PDCD4* mRNA, we did polysome analysis of the MCF7–EGFP–miR-21 and MCF7–EGFP cell lines in absence and presence of La overexpression (Fig. 2*G*). *PDCD4* mRNA was mostly present in the heavier polysomal fractions (48.15% in fractions 5, 6, and 7) in the MCF7–EGFP cells but was shifted to the lighter nonpolysomal fractions (62.73% in fractions 1, 2, and 3) in the MCF7–EGFP–miR-21 cells (Fig. 2*G*, upper panels, blue and green boxes; lower panels, blue and green lines). However, overexpression of La caused the *PDCD4* mRNA to shift back to the polysomal fractions (61.57% in fractions 5, 6 and 7) (Fig. 2*G*, upper panel, purple box; lower panels, purple line), demonstrating that La reversed the translation repression of *PDCD4* mRNA by miR-21. *β-actin* mRNA was mostly associated with heavier polysomal fraction in all the cells.

### La binding site proximal to the miR-21 binding site in *PDCD4* 3ʹUTR is sufficient to reverse miR-21–mediated repression of *PDCD4* mRNA translation

**Figure 3:**
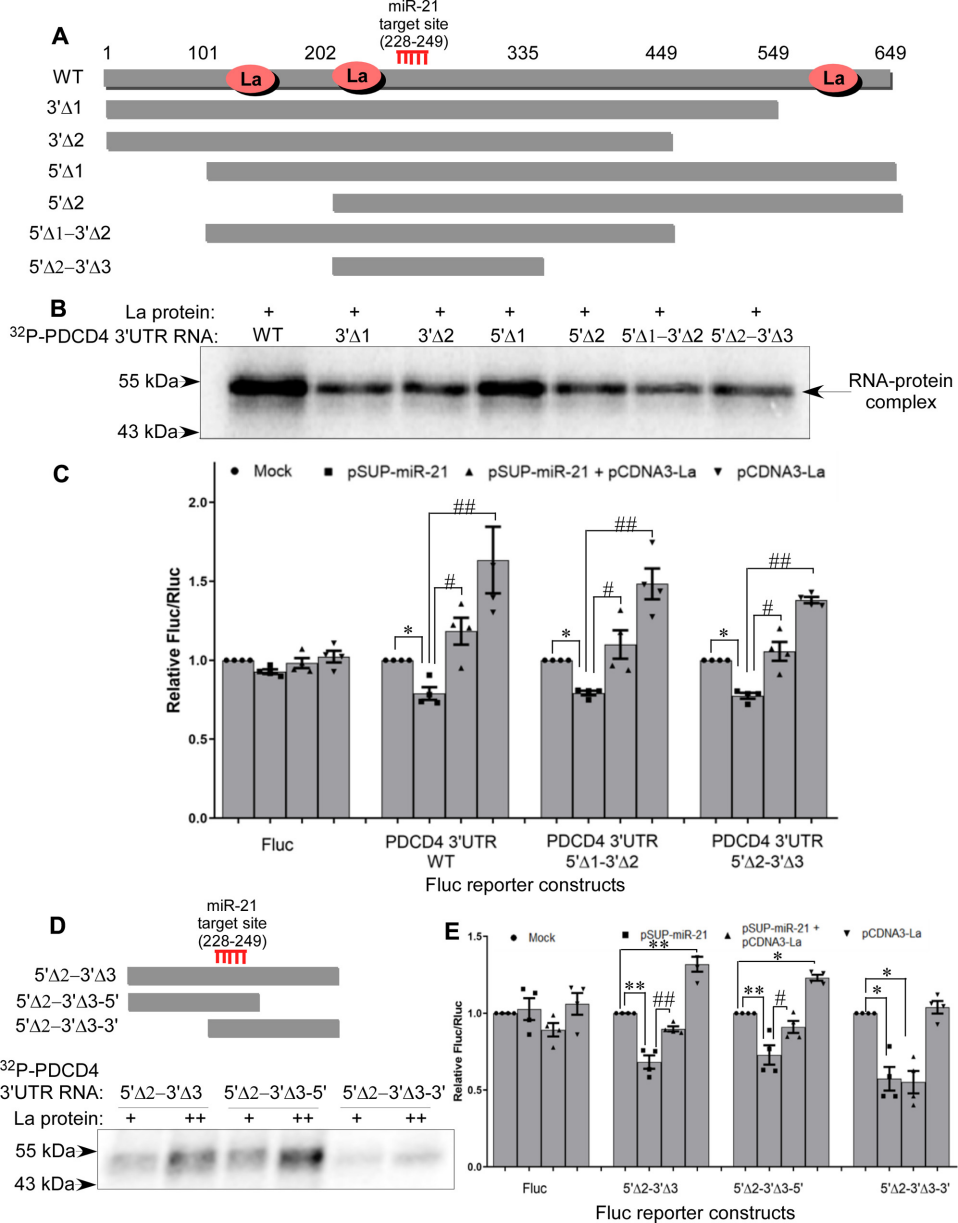
**La binds to a site proximal to the miR-21 binding site in *PDCD4* 3ʹUTR and reverses miR-21-mediated repression of *PDCD4* translation**. *A*, schematic diagram of full-length (FL) *PDCD4* 3ʹUTR and various deletion constructs from the 5ʹ- and 3ʹ-ends. The miR-21 target site (nt 228–249) as well as the La-binding sites, as determined from the RNA–protein interaction studies (below), are indicated. *B*, ^32^P-UTP labeled full-length and various deletion mutants of *PDCD4* 3′-UTR RNA were incubated with purified La protein (20 pmole), UV-crosslinked, digested with RNase A, and the RNA-protein complexes resolved on 10% SDS–PAGE. *C*, luciferase assay of MCF7 cells transfected with reporter gene constructs containing full-length and different deletion mutants of *PDCD4* 3ʹUTR, co-transfected with pSUPER–miR-21 (250 ng) and pCDNA3–La (250 ng) or both. Fluc values are normalized to Rluc values as transfection control. ∗ represents significant difference (*p*-value ≤ 0.05) from respective mock-transfected controls, # and ## represents significant difference (*p*-value ≤ 0.05 and ≤0.01, respectively) from miR-21 transfected sets. *D*, schematic diagram of deletion constructs of the 5ʹΔ2–3ʹΔ3 fragment of the *PDCD4* 3ʹUTR RNA having miR-21 target site (nt 228–249) and two deletions from the 5ʹ- and 3ʹ-ends. ^32^P-UTP labeled RNA of 5ʹΔ2–3ʹΔ3 RNA, and its deletion mutants were incubated with two concentrations (10 and 20 pmole) of purified La protein, UV-crosslinked, digested with RNase A, and resolved on 10% SDS–PAGE. *E*, luciferase assay of MCF7 cells transfected with reporter gene constructs containing 5ʹΔ2–3ʹΔ3 fragment of *PDCD4* 3ʹUTR and its two deletion mutants, co-transfected with pSUP-miR-21 (250 ng) and pCDNA3-La (250 ng) or both. Fluc values are normalized to Rluc values as transfection control. ∗ and ∗∗Represent significant difference (*p*-value ≤ 0.05 and ≤0.01 respectively) from respective mock-transfected controls. # and ## represent significant difference (*p*-value ≤ 0.05 and ≤0.01 respectively) from miR-21 transfected sets. La, lupus antigen; PDCD4, programmed cell death 4; 3ʹUTR, 3ʹ-untranslated region.

To delineate the mechanism by which La antagonizes miR-21–mediated inhibition of PDCD4 translation, we tried to identify the La binding site(s) in the *PDCD4* mRNA 3ʹUTR. RNA-protein UV-crosslinking assay was carried out with different deletion mutants of PDCD4 3ʹUTR RNA, deleted systematically from 5ʹ and 3ʹ ends (Fig. 3*A*), with purified La protein. Deletion of 100 nucleotides from the 3ʹ end (3ʹΔ1) reduced but did not totally abrogate, La binding (Fig. 3*B*). La binding was not reduced on further deletion of 100 nucleotides from the 3ʹ end (3ʹΔ2). Removal of 100 nucleotides from the 5ʹ end (5ʹΔ1) did not affect La binding, but deletion of 200 nucleotides from the 5ʹend (5ʹΔ2) reduced the binding. Removal of 100 nucleotides from the 5ʹ end and 200 nucleotides from the 3ʹend (5ʹΔ2-3ʹΔ3) still allowed La binding, indicating the presence of La binding sites within this region. Finally, the construct lacking 200 nucleotides from the 5ʹ end and 300 nucleotides from the 3′ end (5ʹΔ2-3ʹΔ3) was still able to bind to La protein. Together, these data suggested the presence of three La binding sites: within the 3ʹ 550 to 650 nucleotides of the 3ʹUTR, within the 5ʹ 100 to 200 nucleotides, and a third binding site within the 200 to 335 nucleotides region of the 3ʹUTR, in close proximity to the miR-21 binding site (Fig. 3*B*).

To check the functional effect of each La binding site on miR-21–directed translation repression, luciferase reporter assay was done by transfecting MCF7 cells with reporter gene constructs containing different deletion mutants of *PDCD4* 3ʹUTR, together with miR-21 and La expressing plasmids. Constructs lacking the 3ʹ distal La binding site and lacking both 5ʹ and 3ʹ distal La binding sites but retaining the middle La binding site were found to prevent miR-21–mediated inhibition of reporter activity, suggesting that the presence of the middle La-binding site proximal to the miR-21 binding site is sufficient to rescue *PDCD4* mRNA translation from miR-21–mediated repression (Fig. 3*C*).

From the above experiments, it is not apparent whether the La binding site proximal to the miR-21 binding site is located upstream or downstream of the latter. Therefore, to further characterize the La binding site vis à vis the miR-21 binding site, we cloned 5ʹΔ2-3ʹΔ3_5ʹ and 5ʹΔ2-3ʹΔ3_3ʹ deletion constructs consisting of nucleotide sequences upstream and downstream of miR-21 target site, respectively. UV-crosslinking studies with RNA of both constructs and La protein showed that La binds upstream of the miR-21 binding site (Fig. 3*D*). This was further corroborated by that fact that La protein could reverse miR-21–mediated repression of reporter activity from the reporter gene construct consisting of the *PDCD4* 3ʹUTR sequence upstream of the miR-21 binding site (Fig. 3*E*) Together, these data showed that the La binding site in the *PDCD4* 3ʹUTR proximal to the miR-21 binding site is located upstream of the miRNA binding site. Also both of the 5ʹ proximal La binding sites are located in close proximity to one of the HuR binding sites which is located within the 5ʹ-100 to 200 nucleotides of the *PDCD4* 3ʹUTR as shown previously (37).

### The inflammatory agonist LPS increases PDCD4 expression by enhancing cellular La protein level

**Figure 4:**
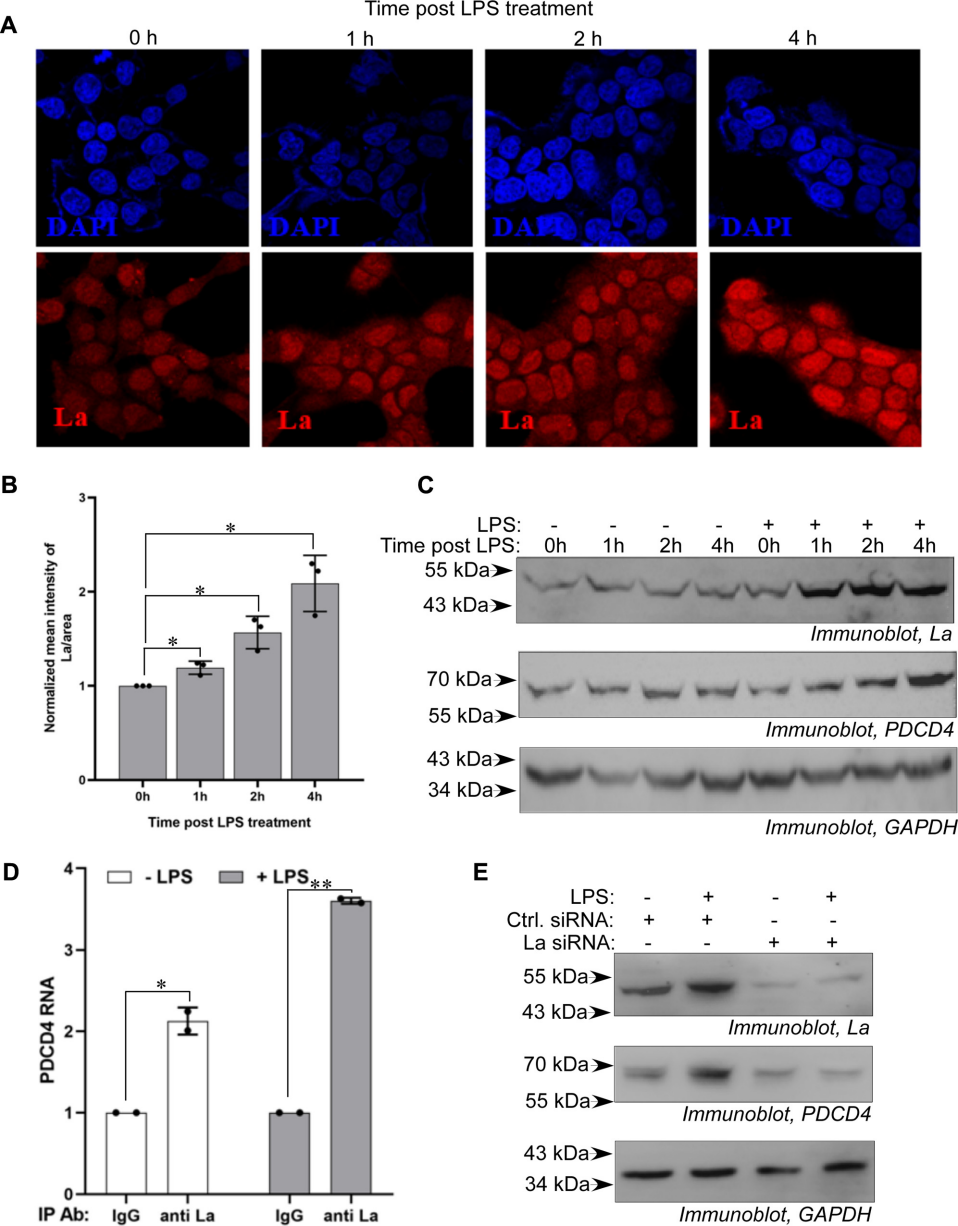
**LPS treatment induces La protein level and enhances PDCD4 expression**. *A*, MCF7 cells were treated with LPS (500 ng/ml), and immunofluorescence of cells collected at various time points after LPS treatment was observed using anti-La primary and AlexaFluor 568-conjugated secondary antibody (*red*). Nucleus was visualized using DAPI staining (*blue*). *B*, fluorescence intensity/cell of three random fields from each time-point after LPS treatment was determined and normalized to 0 h time point and plotted. The data represent mean fluorescence intensities ±SD from three independent experiments. ∗ represents significant difference (*p*-value ≤ 0.05) from 0 h LPS-treated cells. *C*, immunoblots of cytoplasmic lysates of MCF7 cells treated without/with LPS (500 ng/ml) and collected at different time-points posttreatment probed with anti-La, anti-PDCD4, and anti-GAPDH antibodies. *D*, cytoplasmic lysates of MCF7 cells treated with/without (500 ng/ml) LPS for 4 h were immunoprecipitated with La antibody or control IgG. mRNA associated with the immunoprecipitates was subjected to qRT–PCR using *PDCD4* or *β-actin* primers. *PDCD4* mRNA level was normalized to *β-actin* mRNA level. The data represent fold excess of normalized *PDCD4* mRNA in La immunoprecipitate over IgG immunoprecipitate. ∗ and ∗∗Represents significant difference (*p*-value ≤ 0.05 and ≤0.01 respectively) from respective IgG immunoprecipitate controls. *E*, immunoblots of lysates from MCF7 cells transfected with La siRNA or control siRNA (50 pmole) and treated with LPS for 4 h, probed with PDCD4, La and GAPDH antibodies. La, lupus antigen; LPS, lipopolysaccharide; PDCD4, programmed cell death 4.

Previous work has shown that the inflammatory agonist bacterial LPS enhances PDCD4 expression by causing nucleocytoplasmic translocation of HuR. As the proximity of La and HuR binding sites in the *PDCD4* 3ʹUTR was suggestive of interplay between La and HuR in regulating *PDCD4* mRNA translation, we investigated the effect of LPS treatment on La protein in MCF7 cells. Immunofluorescent staining showed the presence of La protein both in the nucleus and cytoplasm of MCF7 cells with a greater abundance in the nucleus. Treatment of MCF7 cells with LPS for 4 h showed a time-dependent increase in the overall level of La both in the nucleus and cytoplasm (Fig. 4, *A–B*). Increased cytoplasmic La protein, after LPS treatment, correlated with the concomitant gradual increase in PDCD4 protein level (Fig. 4*C*). RNA-immunoprecipitation from cell lysate treated with LPS for 4 h showed increased interaction of *PDCD4* mRNA with La protein compared with untreated cells (Fig. 4*D*). siRNA-mediated knockdown of La strongly reduced PDCD4 expression in the cells compared with control siRNA (Fig. 4*E*). LPS treatment for 4 h caused an increase in PDCD4 level in the control siRNA-transfected cell but not in the cells transfected with La siRNA (Fig. 4*E*). Together, these data suggested that LPS-induced expression of PDCD4 in MCF7 cells is mediated, at least in part, by enhancing cellular La level.

### La and HuR function cooperatively to reverse miR-21–mediated translation repression of PDCD4

**Figure 5:**
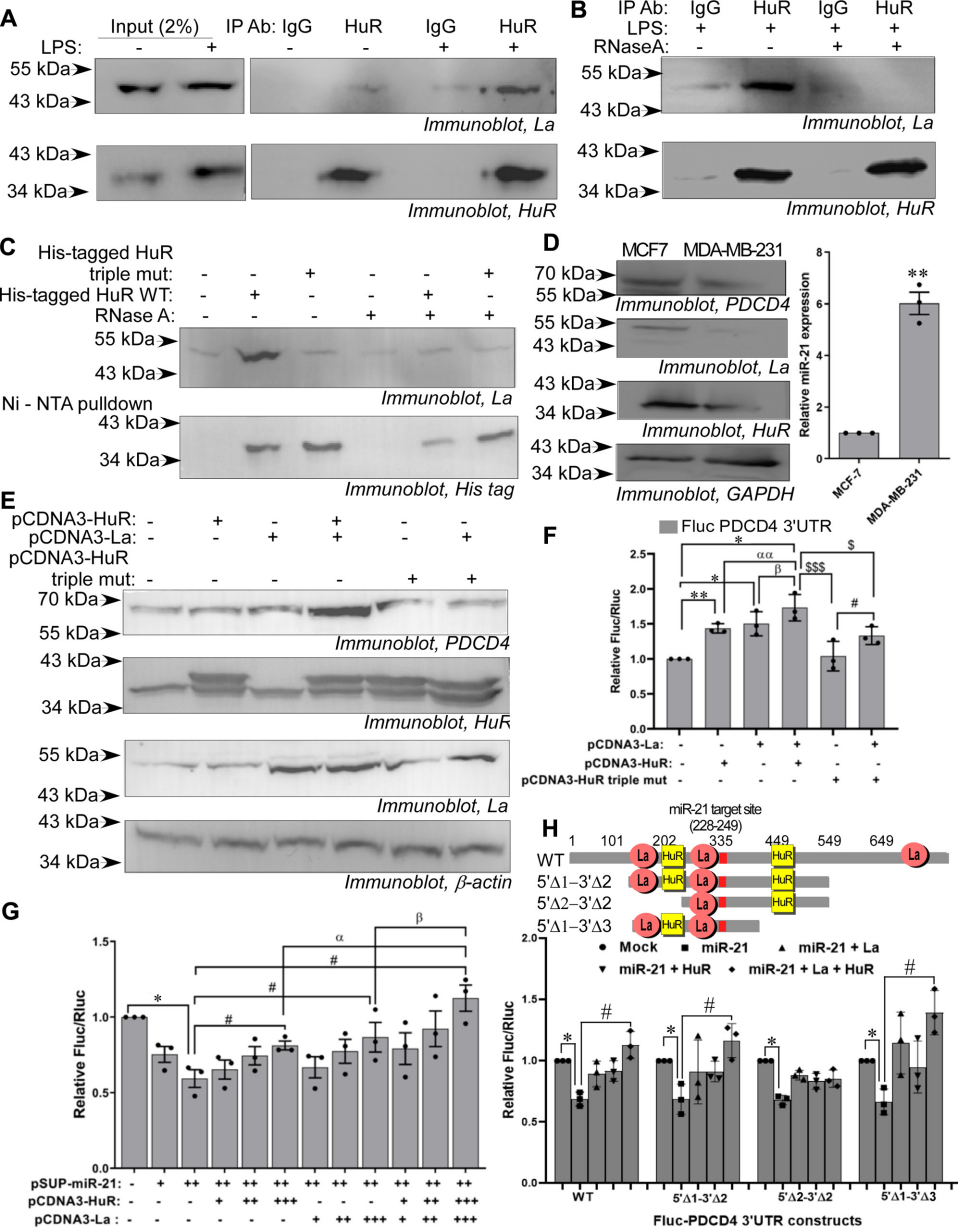
**La and HuR act cooperatively to reverse miR-21-mediated translation repression of PDCD4**. *A*, MCF7 cytoplasmic lysates treated with/without 500 ng/ml LPS for 4 h were immunoprecipitated with anti-HuR or control IgG antibodies and immunoblotted with anti-La and anti-HuR antibodies. The left panel represents immunoblots of 2% of the input lysates. *B*, MCF7 cytoplasmic lysates treated with 500 ng/ml LPS for 4 h were treated with/without RNase A and immunoprecipitated with anti-HuR or control IgG antibodies and immunoblotted with anti-La and anti-HuR antibodies. *C*, MCF7 cells were transfected with 2 μg of constructs expressing His-tagged wild-type HuR or a HuR triple mutant (N21A_Y109A_R147A) incapable of binding PDCD4 RNA. His-tagged proteins were pulled down by Ni-NTA beads, treated without/with RNase A, and immunoblotted using anti-La and anti-His antibodies. *D*, immunoblots of lysates of MCF7 and MDA-MB-231 cells using anti-PDCD4, anti-La, anti-HuR, and anti-GAPDH antibodies. miR-21 expression level in the two cells lines was checked by qRT-PCR from total RNA. ∗∗ represents significant difference (*p*-value ≤ 0.01) from MCF7 cells. *E*, immunoblot of cell lysates transfected either with pCDNA3-La (4 μg) or pCDNA3-HuR (4 μg) or both (2 μg each), or pCDNA3-La and pCDNA3-HuR triple mutant (2 μg each) using anti-PDCD4, anti-HuR, anti-La, and anti-GAPDH antibodies. The two bands in anti-HuR blot indicate myc-tagged and endogenous HuR protein. *F*, luciferase reporter assay of MCF7 cells co-transfected with FlucPDCD4 3ʹUTR reporter gene construct together with pCDNA3-La (400 ng) or pCDNA3-HuR (400 ng) or both (200 ng each) or pCDNA3-La and pCDNA3-HuR triple mutant (200 ng each). Fluc values were normalized to Rluc as transfection control. ∗ represents significant difference from untransfected controls (*p*-value ≤ 0.05). αα (*p*-value ≤ 0.01) and β (*p*-value ≤ 0.05) represent significant difference with cells expressing HuR and La respectively. $ and $$$ represent significant difference (*p*-value ≤ 0.05 and ≤0.005 respectively) with cells expressing both HuR and La, and # represents significant difference (*p*-value ≤ 0.05) with cells expressing HuR triple mutant. *G*, luciferase Reporter assay of MCF7 cells co-transfected with Fluc-*PDCD4* 3ʹUTR reporter gene construct together with pSUPER-miR-21 in two increasing concentrations (100 ng and 200 ng). The cells were co-transfected with pCDNA3-La or pCDNA3-HuR or both in three increasing concentrations (50 ng, 100 ng, and 200 ng), in presence of the higher concentration of pSUP–miR-21 (200 ng). Sum of the amounts of pCDNA3-La and pCDNA3-HuR DNA transfected was same (50 ng, 100 ng, and 200 ng) as when the DNAs were transfected individually. Fluc values were normalized to Rluc as transfection control. ∗ represents significant difference (*p*-value ≤ 0.05) from untransfected controls, # represents significant difference (*p*-value ≤ 0.05) with miR-21 expressing cells, α and β represent significant difference (*p*-value ≤ 0.05) with cells expressing highest concentrations of HuR and La, respectively. *H*, schematic diagram of *PDCD4* 3ʹUTR full length RNA and deletion constructs containing various combinations of La and HuR binding sites used for the reporter assay. Luciferase assay of MCF7 cells transfected with reporter gene constructs containing full-length and various deletion mutants of *PDCD4* 3ʹUTR co-transfected with pSUP-miR-21 and pCDNA3-La or pCDNA3-HuR individually (200 ng each) or in combination (100 ng each for pCDNA3-La and pCDNA3-HuR). ∗ represents significant difference (*p*-value ≤ 0.05) from respective mock-transfected controls, and # represents significant difference (*p*-value ≤ 0.05) from miR-21–transfected sets. HuR, human antigen R; La, lupus antigen; PDCD4, programmed cell death 4; 3ʹUTR, 3ʹ-untranslated region.

As LPS was found to cause nuclear–cytoplasmic translocation of HuR and enhance cellular La level following the same time course, we investigated the presence of interaction between HuR and La proteins in the cytoplasm. Cytoplasmic level of both HuR and La proteins was enhanced after 4 h of LPS treatment (Fig. S5). Immunoprecipitation of HuR from cytoplasmic lysates of LPS-treated cells showed significant enhancement of La protein compared with untreated cells, suggesting an LPS-induced interaction between the two proteins (Fig. 5*A*). We checked whether this interaction is direct or RNA-dependent and found that the interaction between La and HuR was abrogated when co-immunoprecipitation was performed in presence of RNase A, suggesting that the interaction was RNA-dependent (Fig. 5*B*). His-tagged wild-type HuR protein (WT) and a triple mutant HuR protein that lacked the ability to bind to *PDCD4* RNA (N21A + Y109A + R147A) (Fig. S6) were overexpressed in MCF7 cells and pulled down in absence and presence of RNase A treatment by Ni-NTA agarose beads. La protein was found to be pulled down only in association with wild-type HuR protein but not with the mutant, and the association was abolished on treatment of the cell lysate with RNase. This showed that the association of HuR and La only happened in the context of HuR binding to the *PDCD4* 3ʹUTR RNA (Fig. 5*C*).

The RNA-dependent interaction between HuR and La suggested the possibility that they might act together in regulating the expression of PDCD4. We compared the level of cytoplasmic La and HuR proteins between MCF7 and MDA-MB-231 cells which have lower level of PDCD4 expression compared with MCF7 cells. Interestingly, both La and HuR were found to be higher in MCF7 compared with MDA-MB-231 cells, whereas miR-21 level was significantly higher in MDA-MB-231 compared with MCF7 cells (Fig. 5*D*). We then overexpressed HuR and La individually and in combination in MCF7 cells and found that both HuR and La enhanced expression of PDCD4, but the enhancement was higher when La and HuR were expressed together (Fig. 5*E*). However, the enhanced expression of PDCD4 was abrogated when La was overexpressed in combination with the triple mutant HuR (Fig. 5*E*). This was further confirmed by luciferase reporter assay where the co-expression of HuR and La significantly increased the activity of the reporter compared with individual expression of HuR and La, but not when La was co-expressed with the triple mutant HuR, suggesting a cooperative effect of the two proteins on *PDCD4* mRNA translation (Fig. 5*F*). In these experiments, when HuR and La were co-expressed, the amount of the two proteins was adjusted to half the amounts (200 ng transfected DNA each) compared with when the proteins were expressed individually (400 ng transfected DNA) to maintain the stoichiometry between the individual proteins and the mRNA and to ensure that the observed effect was not because of the enhanced binding of one of the proteins only. This resulted in an additive increase in *PDCD4* reporter activity when the proteins were co-expressed. Absence of cooperativity would have shown an expression level of PDCD4 commensurate with expression induced by either HuR or La alone. To confirm the cooperative effect, we also co-expressed HuR and La in amounts equal to their individual expression levels (250 ng each) and found a synergistic increase in luciferase activity from the *PDCD4* 3ʹUTR reporter construct (Fig. S7). There was a 25% increase in reporter gene activity on La overexpression and 30% increase on HuR expression; however, there was more than 100% increase on expressing both La and HuR, which was more than the additive effect of binding of each protein alone. This confirmed the cooperative effect of HuR and La on *PDCD4* mRNA translation.

We then investigated whether there was any cooperative effect of La and HuR in mitigating the miR-21–mediated translation repression of PDCD4. Both HuR and La showed a dose-dependent rescue of Fluc–*PDCD4*-3ʹUTR reporter gene activity, but the increment in reporter activity was found to be significantly more pronounced when both HuR and La were simultaneously overexpressed (Fig. 5*G*). This suggested that cooperative interplay between HuR and La also resulted in a more efficient reversal of miR-21–mediated translation repression of PDCD4. We further confirmed this by transfecting La siRNA in combination with HuR expression and HuR siRNA in combination with La expression, in presence of miR-21 overexpression. The rescue of PDCD4 expression and of *PDCD4* 3ʹUTR reporter construct activity, observed on co-expression of HuR and La, was abrogated in both cases (Figs. S8 and S9), demonstrating the cooperative effect of the two proteins in mitigating miR-21–mediated translation repression of PDCD4.

To investigate which of the binding sites of HuR and La in *PDCD4* 3ʹUTR are required for the cooperative effect of the two proteins, a reporter assay was done with full-length *PDCD4* 3ʹUTR and three different deletion mutants. One of the mutants (5ʹΔ1-3ʹΔ2) contained the HuR and La binding sites proximal to the miR-21 binding site and the distal HuR binding site, the second (5ʹΔ2-3ʹΔ2) contained only one of the proximal La binding sites and the distal HuR binding site, and the third (5ʹΔ1-3ʹΔ3) contained just the proximal HuR and La binding sites. The cooperative effect between HuR and La was only observed in the presence of the HuR binding site and the two La binding sites proximal to the miR-21 binding site (Fig. 5*H*). This observation suggested that HuR and La binding to the *PDCD4* 3ʹUTR in close proximity to each other and the miR-21 binding site allowed their cooperative effect in mitigating miR-21–mediated translation repression of PDCD4.

### La and HuR bind cooperatively to the *PDCD4* 3ʹUTR RNA

**Figure 6:**
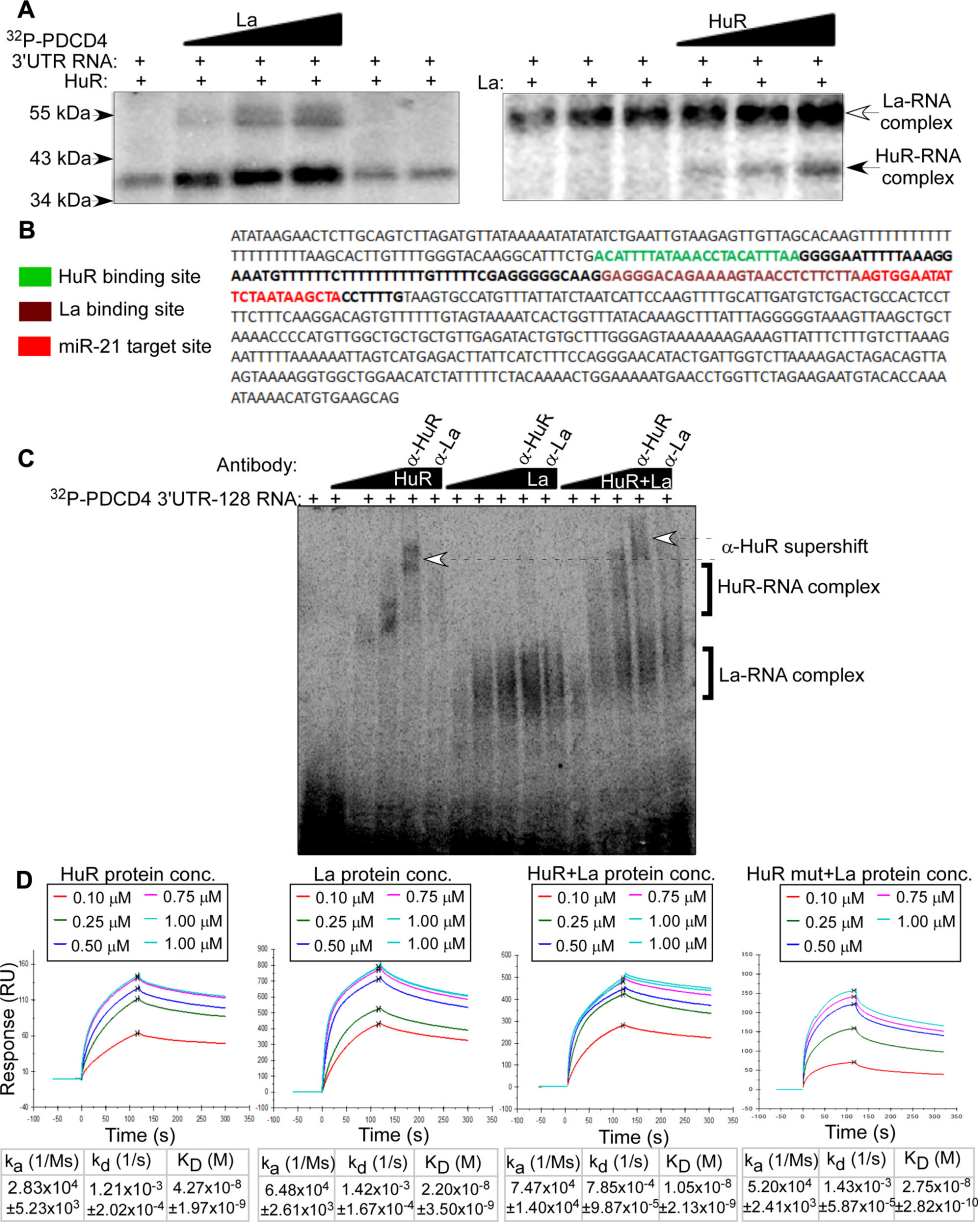
**La and HuR bind cooperatively to *PDCD4* mRNA 3ʹUTR**. *A*,^32^P-UTP-labeled *PDCD4* 3ʹUTR RNA was incubated with either a constant amount (20 pmole) of purified HuR protein in presence of increasing amounts (5, 10, and 20 pmole) of purified La protein (left panel) or with a constant amount of La protein (20 pmole) in presence of increasing amounts (5, 10, and 20 pmole) of HuR protein (right panel). The RNP complexes were UV-crosslinked, digested with RNase A, and resolved on 10% SDS-PAGE. Empty and filled arrows indicate the La and HuR RNP complexes, respectively. *B*, *PDCD4* 3ʹUTR sequence showing the 128 nt sequence (in *bold*) used for the REMSA containing the HuR binding site (*green*), La binding site (*brown*), and miR-21 binding site (*red*). *C*, RNA electrophoretic mobility shift assay (REMSA) using purified HuR and La proteins in three increasing concentrations (5, 10, and 20 pmole), either individually or in combination, with equal concentration of P^32^-labeled-*PDCD4-*3ʹUTR RNA 128 nt fragment shown in B. α-HuR and α-La lanes indicate reaction mixture having HuR and La antibody respectively for supershift assay. The shifted HuR-RNA and La-RNA complexes are indicated by second brackets, and complexes super-shifted with anti-HuR antibody are indicated by *dotted arrows*. *D*, 3ʹ-biotinylated *PDCD4* 3ʹUTR RNA was immobilized on the Biacore SA chip. Increasing concentrations of purified La and wildtype and mutant HuR proteins, either individually or together, were flown over the chip, and response units was plotted against time. The total amount of protein either flown individually, or when together, was same. The curves were fitted considering 1:1 binding kinetics, and binding constants (K_a_, K_d_, and K_D_) were calculated. The binding constants represent the mean ± SEM from three independent experiments. HuR, human antigen R; La, lupus antigen; PDCD4, programmed cell death 4; 3ʹUTR, 3ʹ-untranslated region.

To decipher the mechanism underlying the cooperative function of HuR and La proteins, we investigated whether they exhibit cooperative binding to the *PDCD4* 3ʹUTR *in vitro*. We performed RNA-protein UV cross-linking of radiolabeled *PDCD4* 3ʹUTR RNA with a constant amount of purified His-tagged HuR protein and increasing concentrations of purified La protein which resulted in increased HuR binding to the RNA (Fig. 6*A*). Conversely, the addition of increasing amount of HuR in the presence of a constant amount of La protein also enhanced the interaction of La with *PDCD4* 3ʹUTR RNA (Fig. 6*A*). These observations suggested that HuR facilitates La protein binding to the *PDCD4* 3ʹUTR RNA and vice versa. Further validation of cooperative interaction of HuR and La proteins with *PDCD4* 3ʹUTR RNA was done by RNA electrophoretic mobility shift assay (REMSA) using a 128 nucleotide fragment of the *PDCD4* 3ʹUTR having a HuR and a La binding site and the miR-21 target site (Fig. 6*B*). The La binding site was determined by our deletion analysis (Fig. 3*D*), whereas the HuR binding site was based on PAR-CLIP data (48) and confirmed by mutational analysis (Fig. S10). HuR and La were used separately and in combination. Both HuR and La binding to the *PDCD4* 3ʹUTR RNA fragment showed a concentration-dependent increase, and the gel retardation on HuR binding suggested multimerization of HuR as reported previously (55, 56). The HuR–RNA complex also showed a supershift in presence of HuR antibody. La protein showed a stronger binding affinity to the *PDCD4* 3ʹUTR, but the La antibody did not show a supershift of the La–RNA complex and rather showed a decrease in binding intensity. As the polyclonal La antibody is against N-terminal La motif and RRM1 of La protein which constitutes the main RNA-binding site of the protein, this indicates that antibody binding to the La protein occludes the RNA binding site and therefore reduces the interaction with the RNA. Presence of HuR and La together clearly showed a higher binding affinity to *PDCD4* 3ʹUTR RNA fragment as compared with the individual proteins, and the RNA–protein complex showed supershift with HuR antibody, with the supershifted complex migrating higher than the HuR–RNA supershifted complex. This indicated that the supershifted complex contained both HuR and La bound to the RNA. Together, these observations directly indicated a cooperative binding between HuR and La proteins and the *PDCD4* 3ʹUTR.

We further compared the binding affinities of HuR and La alone, and together, to the *PDCD4* 3ʹUTR RNA by a surface plasmon resonance-based assay. Binding kinetics of La and HuR alone, and in combination, to immobilized *PDCD4* 3ʹUTR RNA showed the average K_D_ of HuR binding to be 4.27 × 10^−8^ M, of La binding to be 2.20 × 10^−8^ M, and of combined La and HuR binding to be 1.05 × 10^−8^ M (Fig. 6*D*). This showed that combined La and HuR binding to the *PDCD4* 3ʹUTR RNA shows an affinity ∼fourfold higher than that of HuR binding alone and ∼twofold higher than La binding alone and therefore demonstrates a cooperative binding of HuR and La to the *PDCD4* 3ʹUTR. The cooperative binding was further confirmed by determining the binding of a combination of La and HuR triple mutant, which showed a K_D_ of 2.75 × 10^−8^ M, significantly higher than that of the combination of HuR and La and nearly equal to the K_D_ of La binding alone.

### La and HuR act cooperatively to reverse miR-21–induced increase in cell proliferation and decrease in apoptosis

**Figure 7:**
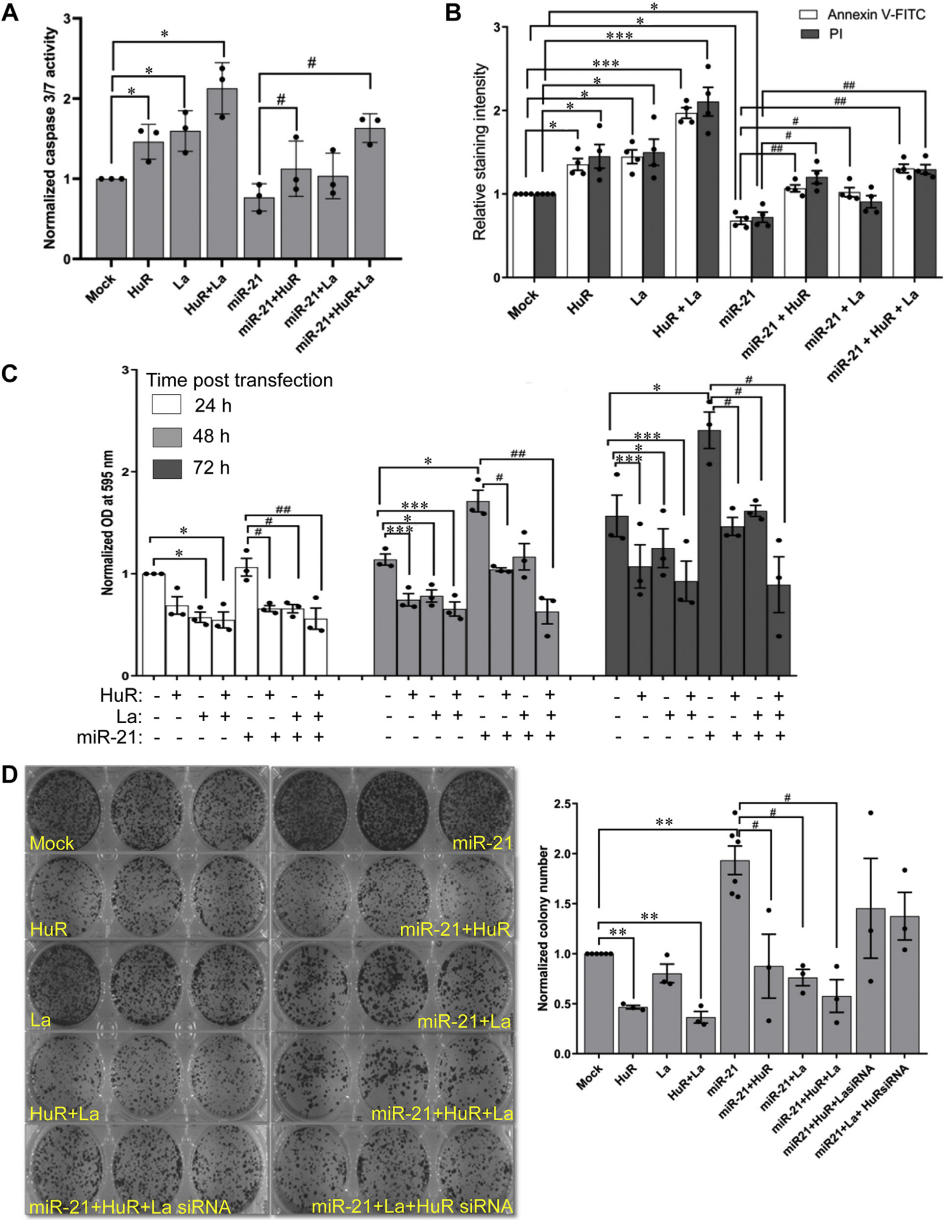
**La and HuR act cooperatively to mitigate miR-21–induced increase in cell proliferation and decrease in apoptosis**. *A*, MCF7 cells were transfected with pCDNA3-HuR, pCDNA3-La, and pSUP-miR-21, individually (2 μg) or in combinations (1 μg each for La and HuR overexpressing constructs). pCDNA3 was used for mock transfection. Posttransfection, MCF7 cells were subjected to serum deprivation for 48 h as an apoptotic stimulus, followed by caspase 3/7 activation assay using a luminescent substrate. Relative light unit values of each sample, representing caspase 3/7 activity, are expressed as fold change from that of mock, taken as 1. ∗ represents significant difference (*p*-value ≤ 0.05) from mock-transfected cells and # represents significant difference (*p*-value ≤ 0.05) from miR-21–transfected cells. *B*, MCF7 cells, transfected with the same combination of DNAs as in A, were serum deprived for 48 h to induce apoptosis. Cells were stained with Annexin V-AlexaFluor 488 and Propidium iodide to detect apoptosis by fluorescent microscopy. Mean fluorescent intensity per cell of Annexin V and propidium iodide for 100 cells per treatment was determined and shown as fold change from mock, taken as 1. ∗, ∗∗, and ∗∗∗ represent significant difference (*p*-value ≤ 0.05, ≤0.01, and ≤0.001 respectively) from respective mock-transfected cells, and # and ## represents significant difference (*p*-value ≤ 0.05 and ≤0.01, respectively) from miR-21–transfected cells. *C*, MCF7 cells were transfected with the same combination of DNAs as in A and B. MTT assay was performed at 24 h, 48 h, and 72 h posttransfection. OD_595_ readings are plotted for each sample as fold change from mock taken as 1. ∗ and ∗∗∗Represent significant difference (*p*-value ≤ 0.05 and ≤0.001 respectively) from respective mock-transfected cells, and # and ## represent significant difference (*p*-value ≤ 0.05 and ≤0.01, respectively) from miR-21–transfected cells. *D*, MCF7 cells were transfected with the same combination of DNAs as in *A*, *B*, and *C*, together with two combinations containing miR-21–expression plasmid (2 μg), La–expression plasmid (2 μg), and HuR siRNA (100 pmole), and miR-21–expression plasmid (2 μg), HuR-expression plasmid (2 μg), and La siRNA (100 pmole). Posttransfection, 1000 cells from each sample were seeded, and after 14 days, colony number was measured by crystal violet staining. Representative image of colony formation experiment is shown in left panel. Colony numbers from each sample are shown as fold change from mock, taken as 1 (right panel). ∗∗ represents significant difference (*p*-value ≤ 0.01) from mock-transfected cells, and # represents significant difference (*p*-value ≤ 0.05) from miR-21–transfected cells. HuR, human antigen R; La, lupus antigen.

PDCD4 is a proapoptotic tumor suppressor protein and the oncogenic activity of miR-21 is partly mediated *via* the downregulation of PDCD4. We have previously demonstrated that HuR can counteract the cell proliferative and antiapoptotic effects of miR-21. Therefore, we investigated whether the cooperativity between La and HuR contributed to the antiproliferative and proapoptotic phenotype. An apoptotic stimulus of 48 h serum starvation was provided to MCF7 cells, overexpressing La and HuR alone or in combination, in presence and absence of overexpressed miR-21. La and HuR were found to independently enhance caspase 3/7 activity, whereas the combination of La and HuR overexpression additively enhanced caspase activity (Fig. 7*A*). The miR-21 overexpression reduced caspase activity, which was partially reversed by HuR and La independently and more significantly reversed when HuR and La were expressed together. Annexin V/PI staining to determine the number of apoptotic cells also indicated that HuR and La overexpression independently enhanced apoptosis, which was further enhanced on combined expression of HuR and La (Figs. 7*B* and S11). The miR-21 overexpression reduced the number of apoptotic cells, which was reversed by independent expression of HuR and La and further reversed on combined expression of the two proteins (Figs. 7*B* and S12). This showed a functional cooperativity between HuR and La in enhancing apoptosis and resisting the antiapoptotic effect of miR-21.

Independent expression of HuR and La also showed reduction in cell proliferation, and a greater reduction when expressed together, as observed over a 72 h period (Fig. 7*C*). HuR and La also reversed the enhanced cell proliferation induced by miR-21, with a significantly higher reversal when both HuR and La were expressed together. Evaluation of colony forming potential of MCF7 cells both in absence and presence of miR-21 overexpression showed that HuR could inhibit colony formation. La overexpression alone did not significantly downregulate colony formation, but a combination of HuR and La overexpression significantly decreased colony formation both in absence and presence of miR-21 overexpression (Fig. 7*D*). Interestingly, siRNA-mediated knockdown of either La or HuR prevented the inhibition of colony formation even if the other protein was overexpressed. These observations show that HuR and La potentiates each otherʹs activity in reducing cell proliferation and colony formation and enhancing apoptosis and therefore cooperatively performs a tumor-suppressive function.

**Figure 8:**
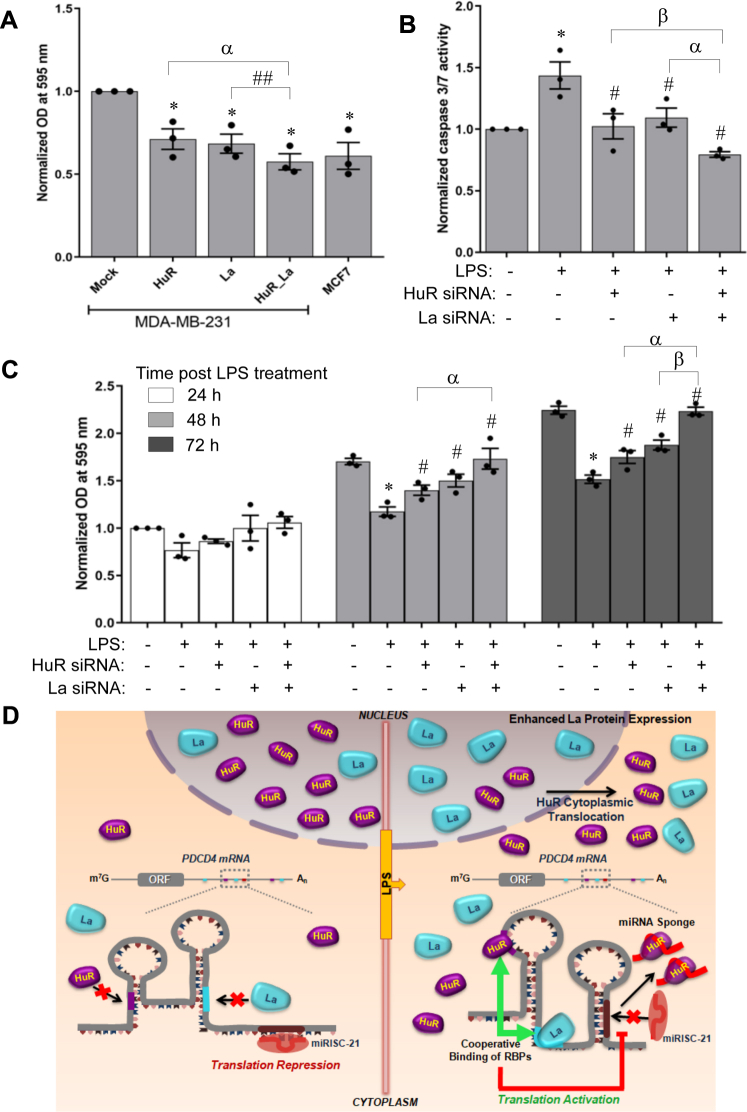
**La and HuR act cooperatively to mediate the proapoptotic and antiproliferative effect of LPS**. *A*, MDA-MB-231 cells were transfected with pCDNA3 (mock) or pCDNA3-HuR and pCDNA3-La, individually (4 μg) or in combination (2 μg each of La and HuR overexpressing constructs). MCF7 cells were transfected with pCDNA3 only. MTT assay was done 48 h post transfection, and OD_595_ readings were plotted as fold change from mock, taken as 1. ∗ represents significant difference (*p*-value ≤ 0.05) from mock-transfected controls. ## (*p*-value ≤ 0.01) and α (*p*-value ≤ 0.05) represents significant difference between indicated groups. *B*, MCF7 cells were transfected with HuR siRNA and La siRNA individually (100 pmole) or in combination (50 pmole each for La and HuR siRNA). Forty-eight hours post transfection cells were treated with/without 500 ng LPS for 4 h followed by 48 h serum starvation. Caspase 3/7 activity was measured using a luminescent substrate. Relative light unit values of each sample, representing caspase 3/7 activity, are expressed as fold change from that of control, taken as 1. ∗ represents significant difference (*p*-value ≤ 0.05) from LPS-untreated controls. # represents significant difference (*p*-value ≤ 0.05) from LPS-treated cells. α and β represents significant difference (*p*-value ≤ 0.05) between indicated groups. *C*, MCF7 cells were transfected and treated as in B. After 4 h of LPS treatment, MTT assay was done at 24 h, 48 h, and 72 h. The OD595 readings are plotted for each sample as fold change from mock taken as 1. ∗ represents significant difference (*p*-value ≤ 0.05) from LPS-untreated controls. # represents significant difference (*p*-value ≤ 0.05) from LPS-treated cells. α and β represent significant difference (*p*-value ≤ 0.05) between indicated groups. *D*, proposed model showing the cooperative effect of La and HuR in reversing the miR-21–mediated translation repression of PDCD4 in response to LPS treatment. HuR, human antigen R; LPS, lipopolysaccharide; PDCD4, programmed cell death 4; RBP, RNA-binding protein.

As the cooperative interplay between La and HuR was found to demonstrate an antiproliferative effect, we checked its effect on MDA-MB-231, an aggressive and highly proliferative breast cancer cell line. MDA-MB-231 cells showed a higher rate of proliferation than MCF7 cells, which decreased on overexpressing La or HuR individually, and was reduced to the level of MCF7 cells on overexpressing both together (Fig. 8*A*). This showed that the cooperative effect of La and HuR could have an antiproliferative effect on a highly proliferative cancer cell line. As LPS had caused the increase in cytoplasmic La and HuR levels, we also checked whether the effect of LPS on cellular physiology was mediated by the cooperative interaction between La and HuR. LPS treatment increased caspase 3/7 activity and reduced the proliferation of MCF7 cells. siRNA-mediated downregulation of La or HuR individually reduced the caspase activity and increased cell proliferation, and combined downregulation of La and HuR further reduced caspase activity and enhanced cell proliferation (Fig. 8, *B*–*C*). Together, these observations showed that La and HuR acted cooperatively to mediate the proapoptotic and antiproliferative effects of LPS on MCF7 breast carcinoma cells.

## Discussion

RBPs regulate gene expression by controlling a number of posttranscriptional processes such as mRNA splicing, stabilization/destabilization, modification, localization, and translation (14, 19, 57). The presence of multiple RBP-binding sites on each mRNA molecule gives rise to the possibility of cooperative interactions between RBPs, which can fine tune the regulatory responses to various stimuli (58, 59). In this study, we have described a cooperative interaction between the RBPs La and HuR in the translation regulation of the proinflammatory tumor suppressor gene PDCD4 in response to the inflammatory agonist LPS. Moreover, HuR and La act cooperatively to mitigate the miR-21–mediated translation repression of PDCD4 and counteract the cell proliferative and antiapoptotic effects of miR-21.

Multiple reports of cooperative interplay between RBPs are known, although nearly no mechanistic details or effects on cellular physiology have been described (60, 61, 62). A classic example of cooperative binding by RBPs is that of the *Drosophila* proteins Pumilio (Pum), Nanos (Nos), and Brain Tumor (Brat) binding to the *hunchback* mRNA in the early embryo (15). The cooperative binding of Pum, Nos, and Brat to two Nanos response elements (NRE2) in the 3ʹUTR of the *hunchback* mRNA allows them to act synergistically to repress the translation of Hunchback protein. The RBP HuR is known to be involved in a number of cooperative interactions with other RBPs. For example, the RBP RNPC1 has been shown to enhance the binding of HuR to the mRNA of the cyclin-dependent kinase inhibitor P21 and acts cooperatively with HuR to enhance the stability of *p21* mRNA (63). This cooperativity is mediated by the direct interaction between the RRM domains of HuR and RNPC1. The RBP TIA-1 enhanced the binding of HuR to the *cytochrome c* mRNA 3ʹUTR under ER stress, but the effects of TIA-1 and HuR on *cytochrome c* mRNA translation was antagonistic rather than synergistic (64). TIA-1 and HuR have also been reported to bind to the *PDCD4* mRNA 3ʹUTR, but the binding appears to be competitive rather than cooperative (38). HuR and PTB also jointly regulate the translation of hypoxia-inducible factor 1α (*HIF1α*) mRNA in response to hypoxia, but cooperativity has not been demonstrated (65). In the study reporting the cooperative binding of TIA-1 and HuR to the *cytochrome c* mRNA 3ʹUTR, the authors did not detect simultaneous binding of HuR and TIA-1 in direct binding assays employing REMSA (64). Positive cooperativity would imply that binding of one protein has a positive impact on binding of the second. In our study, simultaneous binding of HuR and La to the *PDCD4* 3ʹUTR has been observed using REMSA, and it has been seen that HuR positively affects La protein binding to the *PDCD4* 3ʹUTR RNA and vice versa. This has been further reinforced using surface plasmon resonance assay which has substantiated a direct cooperative binding of HuR and La to the *PDCD4* 3ʹUTR. Therefore, a cooperative effect between HuR and La in the regulation of translation of *PDCD4* mRNA, both at the level of direct RNA binding, and functionally at the level cellular behavior, is observed in this study.

As the interplay between RBP-mediated and miRNA-mediated posttranscriptional regulatory processes has become more evident, there have been multiple reports of interactions between RBPs that regulate mRNA translation/turnover and RBP components of the miRNA-induced silencing complex (miRISC) (20, 22, 66). For example, the binding of the RBP AUF1 to mRNAs is cooperative or competitive with AGO2, the main RBP component of the miRISC, in an mRNA-specific manner and allows transcript-specific combinatorial binding which can regulate mRNA degradation in a coordinated manner (67). The translation regulating protein Pumilio (PUM) has also been shown to act cooperatively with AGO2 to repress the translation of mRNAs such as cyclin-dependent kinase inhibitor 1B (*CDKN1B/p27*) and *E2F3* (66, 68). However, PUM1 and PUM2 have also been found to act antagonistically with AGO2 in the case of a subset of mRNAs, in which case PUM binding inhibits AGO2 binding and prevents miRNA-mediated mRNA degradation (69).

The functional interplay between specific RBPs and miRNAs in regulating translation and/or stability of multiple mRNAs is increasingly apparent as a common mode of posttranscriptional regulation in different physiological and pathological conditions (20, 21, 70). This interplay can be competitive, such as in the case of HuR inhibiting the translation repression of the *p53* mRNA by miR-125b during genotoxic stress or DND1 preventing the miR-221/miR-222–mediated repression of CDKN1B (71, 72). It can also be cooperative, such as in the case of the RBP tristetraprolin facilitating the miR-16–mediated translation repression of tumor necrosis factor and cycloxygenase 2 mRNAs or HuR cooperating with miRNA let-7 to repress the translation of *c-myc* mRNA (73, 74). However, it is not clear whether in such cases the cooperative effect arises from direct cooperativity between binding of the RBP and any component of the miRISC complex to the target mRNA or *via* direct interaction between the RBP and the miRNA (20). This interplay between miRNA and RBP-binding can be made further fine-tuned by involving multiple RBPs. In the present study, it is the cooperative binding of two RBPs, HuR and La, to a common mRNA target, which can inhibit the miRNA-mediated translation repression. This is possibly the first report of two RBPs acting synergistically to antagonize miRNA-mediated translation repression. Our observations demonstrate that HuR and La can reciprocally enhance the binding of each other to the *PDCD4* mRNA, and although each can independently counteract miR-21–mediated translation repression of PDCD4, they exert a synergistic effect in reversing the translation repression when they bind cooperatively to the *PDCD4* mRNA. This adds a further layer of complexity to the combinatorial regulation of mRNA translation/turnover by RBPs and miRNAs in complex regulatory environments.

Another interesting observation of this study is that a single stimulus, the inflammatory agonist LPS, regulates the function of two different RBPs, HuR, and La, in different ways. While LPS has been shown earlier to cause the nuclear cytoplasmic translocation of HuR, in this study, it was found to induce an increase in both nuclear and cytoplasmic La level. Together, these two processes result in increased cytoplasmic HuR and La concentrations, leading to cooperative binding to the *PDCD4* mRNA. To the best of our knowledge, this phenomenon of a single stimulus inducing two RBPs in different ways, which then act cooperatively to regulate mRNA translation, is unprecedented. Therefore, we propose a model in which LPS causes the nuclear cytoplasmic translocation of HuR and simultaneously causes increased expression of La. Resultantly, cytoplasmic HuR and La bind cooperatively to the *PDCD4* mRNA 3ʹUTR and prevent the binding of miR-21 by causing an RNA structural switch, thereby reversing translation repression. The free miR-21 is simultaneously sponged up by HuR, causing further dissociation of miR-21 from the *PDCD4* mRNA 3ʹUTR, resulting in enhanced translation (Fig. 8*D*). Elucidation of the signal transduction pathways triggered by LPS, which cause the simultaneous induction of cytoplasmic HuR and La proteins by two different mechanisms, will provide further insights into this complex regulatory network.

## Experimental procedures

### Plasmid constructs

Human *PDCD4* 3ʹ-UTR (642 nt) was cloned into pCDNA3.1 and pCDNA3-FLuc vector downstream of firefly luciferase (Fluc) gene as described earlier (37). Different deletion constructs of the 3ʹUTR were generated by PCR and cloned into pCDNA3.1. Double-stranded DNA oligo encoding wildtype miR-21 sequence was cloned into pSUPER vector which is transcribed to produce miR-21 shRNA. HuR and La were expressed from the mammalian expression vector pCDNA3.1 and from the bacterial expression vector pET28b for HuR and pRSET for La (pCDNA3-La and pRSET-La plasmids were a kind gift from S. Das, IISc, Bangalore). Triple mutant HuR (N21A + Y109A + R147A) was cloned by megaprimer-based site-directed mutagenesis.

### Cell culture, treatment, and transfection

MCF7 human breast carcinoma cells and MDA-MB-231 cells were maintained in Dulbeccoʹs Modified Eagle Medium (Thermo Scientific) supplemented with 10% fetal bovine serum. Cells were treated with 500 ng/ml LPS from *E. coli* (Sigma-Aldrich). Cells were transfected with plasmid vectors, siRNA, or antagomiRs using Lipofectamine 2000 (Thermo Scientific) in serum-free OptiMEM or low glucose DMEM media. pCDNA3.1 plasmid and control oligos were used as transfection normalization control. pRL–CMV was transfected as normalization control for Fluc containing plasmids. miR-21/EGFP and EGFP-expressing stable MCF7 cell lines were generated as described earlier (37).

### RNA-protein UV-crosslinking assay

*In vitro*-transcribed α-^32^P-UTP-labeled RNAs of equal specific activity (∼100,000 cpm = 15 fmol) were incubated with protein samples (MCF7 cytoplasmic extract, purified HuR, and La proteins as indicated in results) in 2× RNA-binding buffer (5 mM HEPES, 25 mM KCl, 2 mM MgCl_2_, 2 mM DTT, 0.1 mM EDTA, 3.8% glycerol, 1.5 mM ATP) having 1 μg/μl yeast tRNA, for 30 min on ice and UV crosslinked by irradiation (500 mJ/cm^2^ radiation for 10 min) in an UV crosslinker. Unbound RNAs were digested by treatment with 30 mg of RNase A at 37 °C for 30 min (75). The RNA–protein complexes were resolved on 12% SDS–PAGE followed by phosphorimaging (Typhoon Trio, GE Healthcare).

### RNA electrophoretic mobility shift assay

The 128 bp *PDCD4* DNA template having T7 promoter at 5ʹ end was generated by PCR, followed by *in vitro* transcription to generate 128 nt α-^32^P-UTP-labeled *PDCD4* 3ʹUTR RNA. Two increasing concentrations of purified HuR and La proteins were incubated with labeled RNA in RNA-binding buffer for 30 min on ice. For supershift assay, anti-HuR and anti-La antibody (0.2 μg each) were added to the reaction mixtures containing highest protein concentration along with the other components. Reaction mixtures was resolved on 6% native PAGE at 4 °C followed by phosphorimaging.

### RNA affinity chromatography followed by mass spectrometry

Monolayer of MCF7 cells was harvested and resuspended in S10 cell lysis buffer (10 mM HEPES pH 7.4, 15 mM KCl, 1 mM PMSF, and 1 mM DTT) and lysed by passing through 26 gauge needle on ice. After centrifugation at 10,000*g* for 20 min at 4 °C, the supernatant was collected and used for the binding reaction. Ten micrograms of 3ʹ-biotinylated *PDCD4* 3ʹUTR RNA was incubated with 500 μg of MCF7 S10 lysate in binding buffer (10 mM HEPES pH 7.0, 50 mM KCl, 10% glycerol, 1 mM EDTA, 1 mM DTT, 0.15 μg/ml yeast tRNA, and 100 u/ml RNase Inhibitor) for 4 h at 4 °C followed by binding reaction with 50 μl of high capacity streptavidin agarose bead (Thermo Scientific) for 2 h at 4 °C. The beads were washed four times with binding buffer containing 200 mM NaCl. The captured proteins were eluted by addition of 500 μl elution buffer containing 1 M NaCl (10 mM HEPES pH 7.0, 50 mM KCl, 10% glycerol, 1 mM EDTA, 1 mM DTT, and 1 M NaCl). The eluted proteins were resolved in 10% SDS-PAGE after concentrating through 3 kDa cut-off centrifugal concentrator (Millipore). The gel was stained with SYPRO Ruby stain (Thermo Scientific). Excised protein bands following SDS- PAGE were digested in gel with trypsin and analyzed by mass spectroscopy. Full MS in the mass range between m/z 375 and m/z 1700 was performed on an Orbitrap Mass analyzer, followed by CID-based MS/MS, performed in the scan range of m/z 100 and m/z 2000. Mascot Distiller software was used to identify the peptides with GG modification, in which the peptide mass tolerance threshold was set to 10 ppm, and the maximum fragment mass tolerance was set between 0.6 Da.

### Immunoblotting

MCF7 or MDA-MB-231 cell lysates were resolved by 12% SDS–PAGE, electrotransferred to polyvinylidene difluoride membrane followed by immunoblotting using antibodies against PDCD4 (Cell Signaling Technology), HuR, La/SSB, and GAPDH (Santacruz Biotechnology) as primary antibodies, and horseradish peroxidase-conjugated β-Actin (Genscript) or anti-mouse or anti-Rabbit (Cell Signaling Technology) as secondary antibodies according to the primary antibodies used. The bands were detected using Femtolucent chemiluminescence detection kit (G-Biosciences).

### Reporter assay

Cells were transfected with specific amounts of firefly luciferase reporter gene constructs with full-length or mutant *PDCD4* 3ʹUTRs and a Renilla luciferase reporter construct as indicated in figure legends. Transfected cells were lysed with passive lysis buffer after 36 h of transfection. Luciferase assay was performed using Dual-Glo Luciferase assay system (Promega) as per manufacturerʹs protocol. Luminescence was measured in a Plate Chameleon V (Hidex) multilabel microplate reader or luminometer (GLOMAX 20/20, Promega).

### RNA immunoprecipitation

Cell lysates were precleared by incubation with swelled Protein A sepharose beads (Sigma-Aldrich) in NT2 buffer (50 mM Tris-Cl pH 7.4, 150 mM NaCl, 1 mM MgCl_2_, 0.05% NP-40). Precleared cell lysate was incubated with specific antibody (1:10 dilution) in NT2 buffer for 4 h at 4 °C. Preswelled Protein A sepharose beads were incubated with the antibody–protein complex for overnight at 4 °C. Unbound proteins were removed by washing five times with 500 μl NT2 buffer. This was followed by RNA isolation from the beads using TRIzol reagent (Thermo Scientific), and cDNA was prepared using MMLV Reverse Transcriptase (Thermo Scientific).

### Co-immunoprecipitation

Immunoprecipitation was performed using Pierce Direct IP kit following manufacturerʹs instructions. Briefly, primary antibodies were crosslinked to the supplied amine beads in coupling buffer supplemented with sodium borohydride for 2 h at room temperature. Unbound antibodies were removed by washing with 1 M NaCl solution. One milligram precleared cell lysate was incubated with the antibody coupled beads for 4 h or overnight at 4 °C. For Co-IP, low stringency wash buffer (NT2 buffer without NP-40) was used for the washing. The bound protein complex was eluted using low pH elution buffer and resolved in 10% SDS-PAGE followed by immunoblotting.

### Ni-NTA co-pull down assay

MCF7 Cells overexpressing His-tagged proteins were lysed in NT2 buffer supplemented with 10 mM imidazole. Cell lysates were incubated with Ni-NTA agarose beads (Thermo Scientific) for 3 h at 4 °C. Nonspecific proteins were removed by washing with NT2 buffer containing 30 mM imidazole. Beads were resuspended in SDS-PAGE gel loading buffer, denatured at 100 °C and resolved in 10% SDS-PAGE followed by immunoblotting.

### mRNA quantification by real-time PCR

Total RNA was isolated from cells using TRIzol Reagent and used for reverse transcription followed by quantitative PCR. RNA quantity normalization between samples for ΔΔC_t_ calculation was done using GAPDH or β-actin primers. qPCR was performed using Power SYBR Green reagent (Thermo Scientific), in Step One plus real-time PCR system (ABI, Thermo Scientific).

### Cell proliferation, colony formation, and apoptosis assays

After 36 h of transfection with plasmid constructs/siRNA, 10^4^ cells were seeded in 96-well plate, and cell proliferation study was done at 24, 48, and 72 h by MTT based assay (Sigma Aldrich). For colony-forming assay, 10^3^ transfected cells were seeded in 6-well plate and allowed to form colonies for 14 days. Colonies were counted after staining with crystal violet. For apoptosis assay, after 36 h of transfection, cells were serum-starved (DMEM with 2% fetal bovine serum) for 48 h. Apoptosis was measured by CaspaseGlo caspase 3/7 assay kit (Promega) and by Annexin V/PI staining using AlexaFluor-488 Annexin V/Dead Cell Apoptosis Kit (Thermo Fisher Scientific). Cells were treated with 500 ng LPS 36 h after transfection for 4 h, following which cell proliferation and apoptosis assays were carried out.

### Immunofluorescence

MCF7 cells treated with LPS for various time points were fixed with 4% paraformaldehyde and treated with 1:20 diluted rabbit polyclonal anti-La/SSB antibody (Santa Cruz Biotechnology) followed by 1:400 diluted Alexafluor 568-conjugated mouse anti-rabbit antibody (Cell Signaling Technology). DAPI (1 ng/ml) was used for nuclear staining. Images were taken using Zeiss LSM 710 scanning confocal microscope.

### Polysome analysis

Polysome analysis was done by ribosomal fractionation following the protocol described earlier (76). In brief, MCF7-EGFP and MCF7-EGFP-miR-21 cells were grown up to 80% confluency in 10 cm dishes in DMEM-10% FBS. The cells were then transfected in serum-free medium. Six hours after transfection, the medium was replaced with 10% FBS-containing medium. Thirty-six hours after transfection, the cells were treated with cycloheximide (0.1 mg/ml) for 30 min before harvesting. The cells were then harvested and washed with ice-cold PBS containing 0.1 mg/ml cycloheximide. The cells were lysed in polysome lysis buffer (200 mM HEPES pH-7.4, 100 mM KCl, 5 mM MgCl_2_, 2 mM DTT, 1× protease inhibitor [Protease Arrest, G Biosciences], and 100 U/ml RNase inhibitor [RiboLock Thermo Fisher Scientific]) by pipetting, and cytosolic extract was obtained by centrifugation at 10,000*g* for 20 min at 4 °C. The extract was carefully overlayed on a 10 to 50% (w/v) sucrose gradient and centrifuged at 100,000*g* for 4 h. Fixed volume fractions were collected using a programmable gradient fractionator (Biocomp), and absorbance of each fraction was measured at 254 nm and plotted against fraction number. RNA was isolated from the fractions by phenol–chloroform extraction and ethanol precipitation, washed with 70% ethanol and dried for further processing.

### Surface plasmon resonance assay

*PDCD4* 3ʹUTR RNA or miR-125b pri-miRNA was biotinylated at 3ʹ end as per manufacturer's protocol (Thermo Scientific Pierce RNA 3ʹ End Biotinylation Kit). Surface Plasmon Resonance assay was done on the Biacore T200 (GE-Healthcare Life Science). Biotinylated RNA was immobilized on the SA-chip (GE-healthcare Life Sciences) by interaction with streptavidin. Five increasing concentrations of purified HuR and La and the equimolar mixture of the two proteins or of La and HuR triple mutant were flown over the chip using HBS-EP buffer and the sensorgrams obtained. Binding constants were calculated by fitting to 1:1 binding kinetics model (Supporting information).

### Statistical analysis

All graphical data represent mean ± standard deviation of at least three independent experiments (biological replicates). ∗, #, $, α, or β signifies a *p*-value ≤ 0.05, ∗∗, $$, or ## signifies a *p*-value ≤ 0.01, ∗∗∗, $$$, or ### signifies a *p*-value ≤ 0.005 (Paired two-tailed Student's *t* test) between controls and samples as indicated in the Figures.

## Data availability

All data described in the manuscript are contained in the manuscript. The mass spectrometry proteomics data have been deposited to the ProteomeXchange Consortium *via* the PRIDE (77) partner repository with the dataset identifier PXD017376.

## Conflict of interest

The authors declare that they have no conflicts of interest with the contents of this article.
