# Supplementary material for: RNA-binding proteins La and HuR cooperatively modulate translation repression of PDCD4 mRNA
Source: J Biol Chem. 2020 Dec 9;296:100154. doi: 10.1074/jbc.RA120.014894 (PMC7949077; doi:10.1074/jbc.RA120.014894)
Supplement: Supplementary data [file mmc1.pdf]

Fig. S1

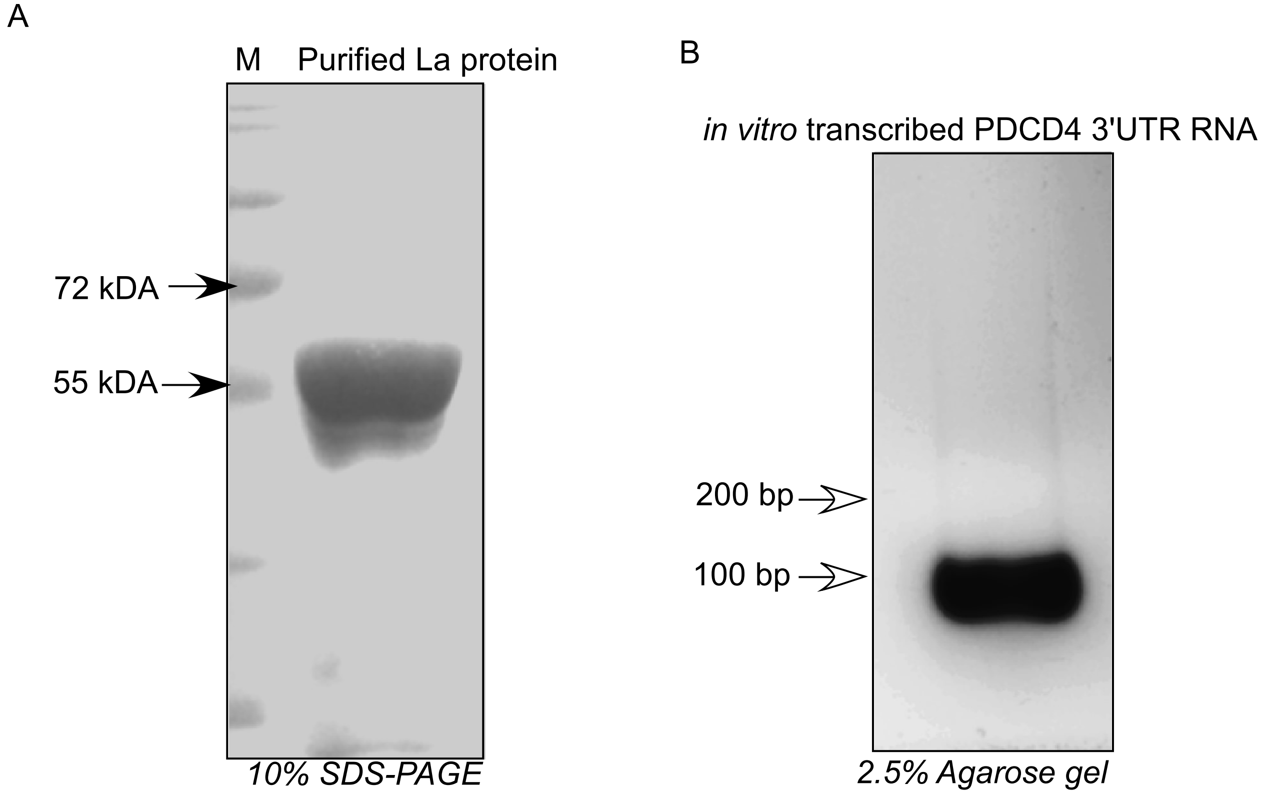

Fig.S1. A. Purified, recombinant His-tagged La protein run on SDS-10% PAGE.
B. in vitro transcribed, purified PDCD4 3'UTR RNA resolved on 2.5% agarose gel.
Open arrows represent double-stranded DNA mol. wt. markers from 100 bp DNA ladder.

Fig. S2

La + miR-125b pri-miRNA

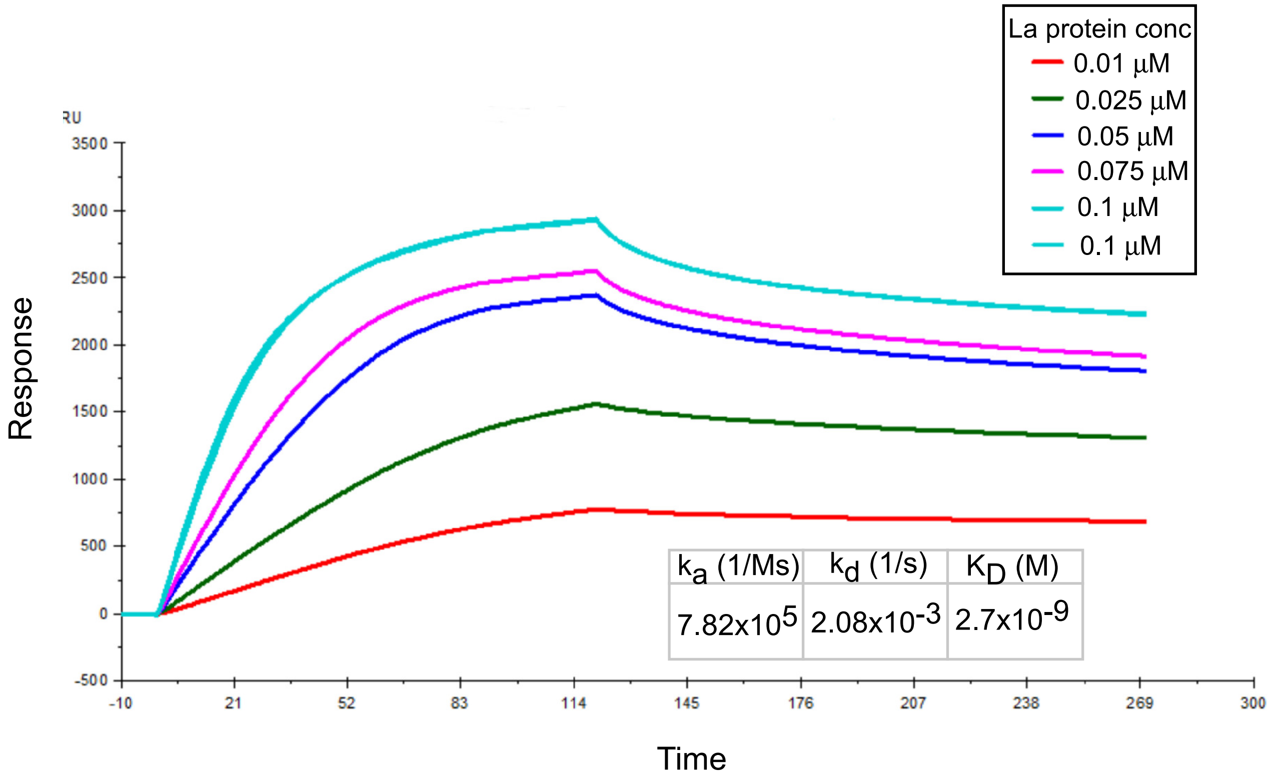

k_a (1/Ms)	k_d (1/s)	K_D (M)
$7.82x10^5$	$2.08x10^{-3}$	$2.7x10^{-9}$

Fig. S2 i*n vitro* transcribed 3’-biotinylated miR-125b pri-miRNA was immobilised on the Biacore SA chip. Increasing concentrations of purified La protein was flowed over the chip and the Response Units were plotted against time. Binding constants (K_a, K_d and K_D) were calculated considering 1:1 binding kinetics.

Fig. S3

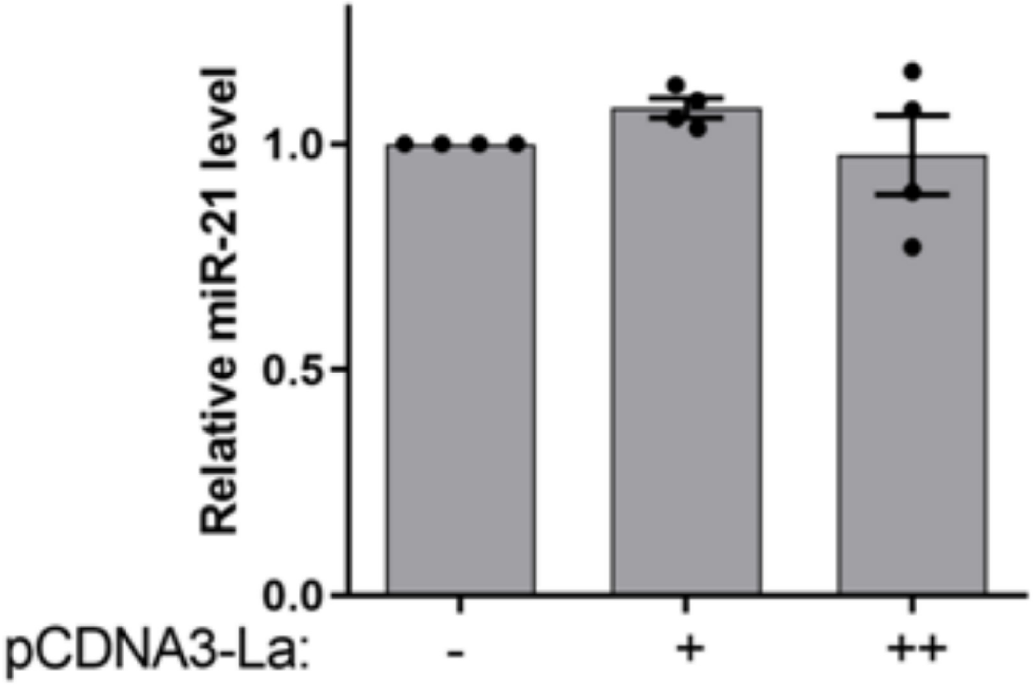

Fig.S3. Relative level of miR-21 expression in MCF7 cells transfected with a La expression construct in two increasing concentrations (2 μg and 4 μg). Data represents Mean +/- SD from four independent experiments.

Fig S4

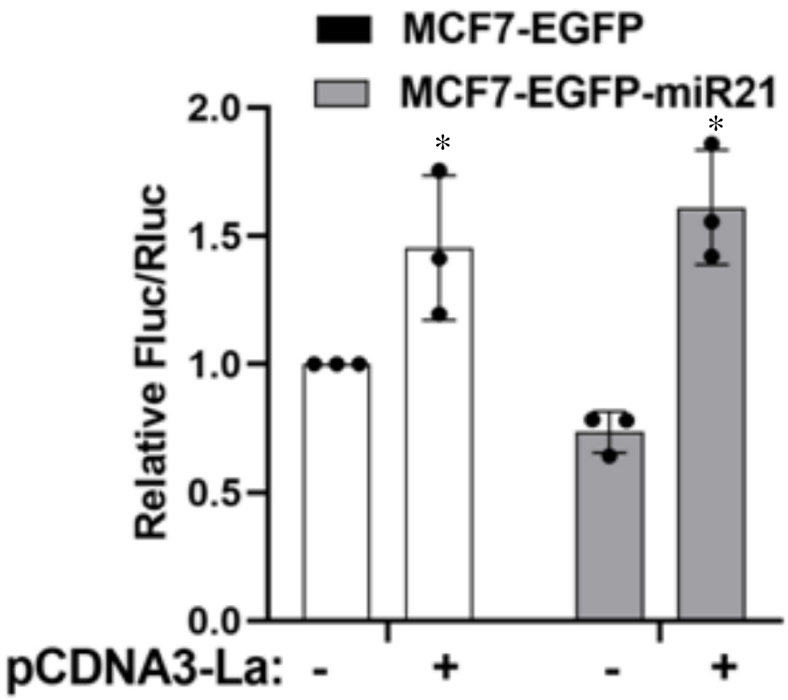

Fig. S4. Luciferase assay of MCF7-EGFP and MCF7-EGFP-miR-21 cell lines co-transfected with Fluc-PDCD4 3'UTR reporter construct and pCDNA3-La (200 ng). pCMV-Rluc was transfected as transfection control. Data represents Mean +/- SD from three independent experiments. * represents significant difference (p </= 0.05) with respective controls.

Fig S5

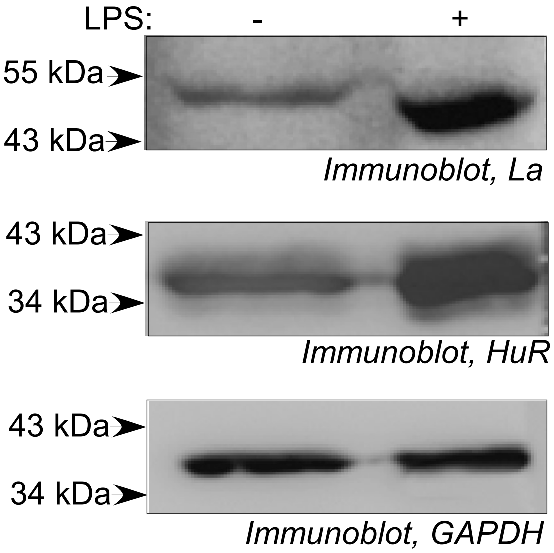

Fig. S5. MCF7 cell cytoplasmic lysates treated without/with 500 ng/ml LPS for 4 hours were immunoblotted with La, HuR and GAPDH antibodies.

Fig S6

A

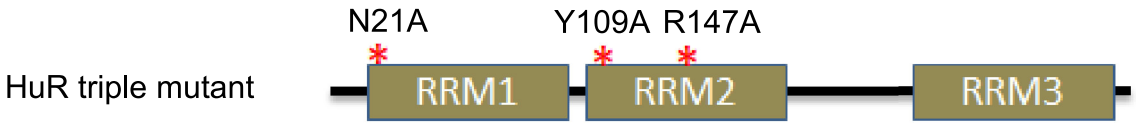

B

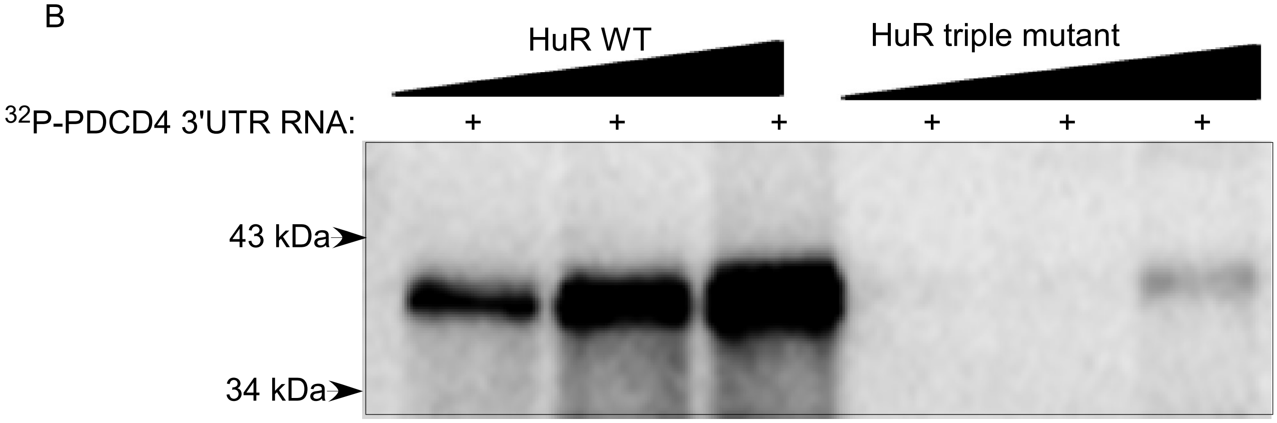

C

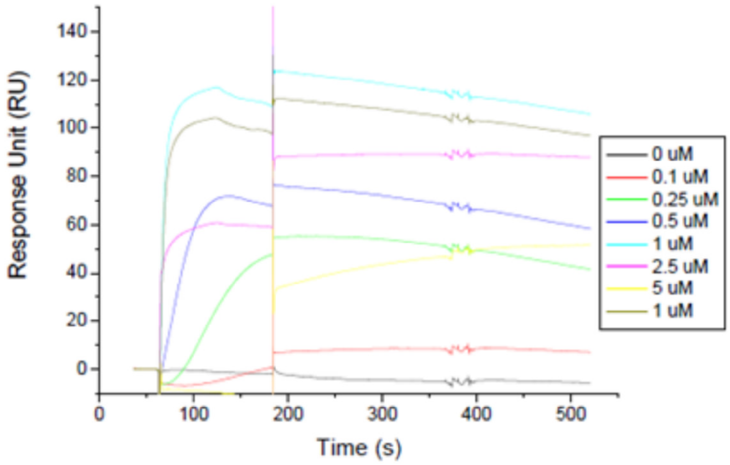

Fig. S6. A. Schematic diagram of HuR protein indicating three amino acid point mutations.

B. *in vitro* binding of ^{32}P-labelled PDCD4 3'UTR RNA with three increasing concentrations (10, 20 and 40 pmole) of purified HuR WT and HuR_N21A_Y109A_R147A mutant proteins.

C. SPR assay of HuR_N21A_Y109A_R147A mutant protein with biotinylated PDCD4 3'UTR RNA. The SPR sensogram shows failure of binding.

Fig S7

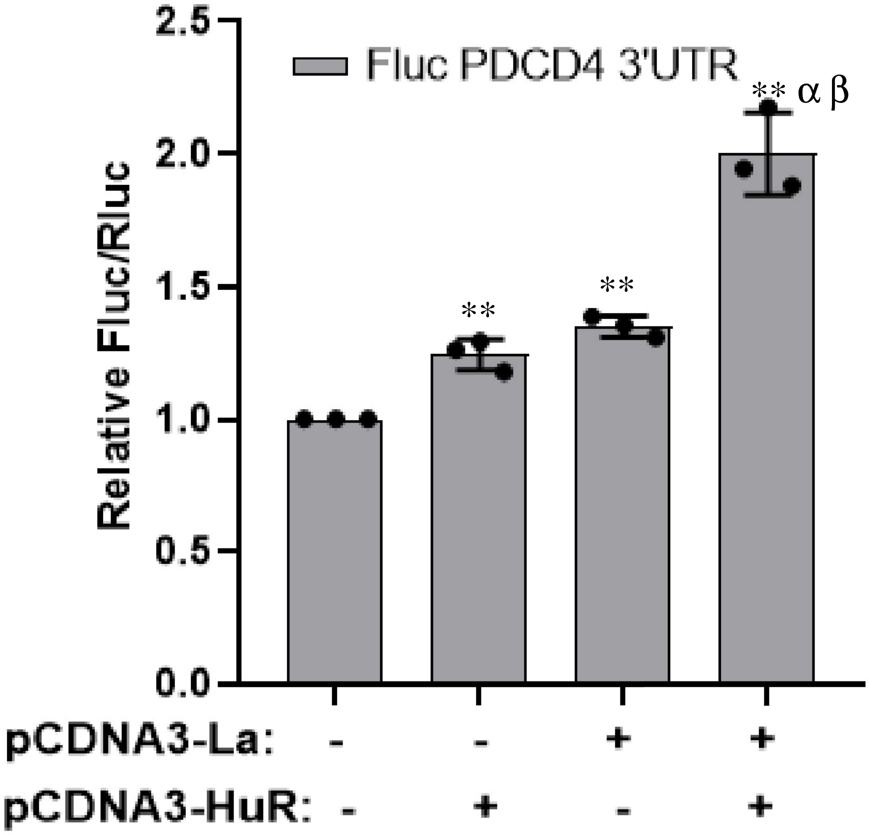

Fig. S7. Luciferase assay of MCF7 cells co-transfected with Fluc-PDCD4 3'UTR reporter construct and pCDNA3-La and pCDNA3-HuR expression constructs. 250 ng of pCDNA3-La and pCDNA3-HuR respectively was transfected either individually or together. pCMV-Rluc was transfected as transfection control. Data represents Mean +/- SD from three independent experiments. ** represents significant difference (p </= 0.01) from mock-transfected control whereas α and β represents significant difference (p </= 0.05) from individual transfection of HuR and La respectively.

Fig S8

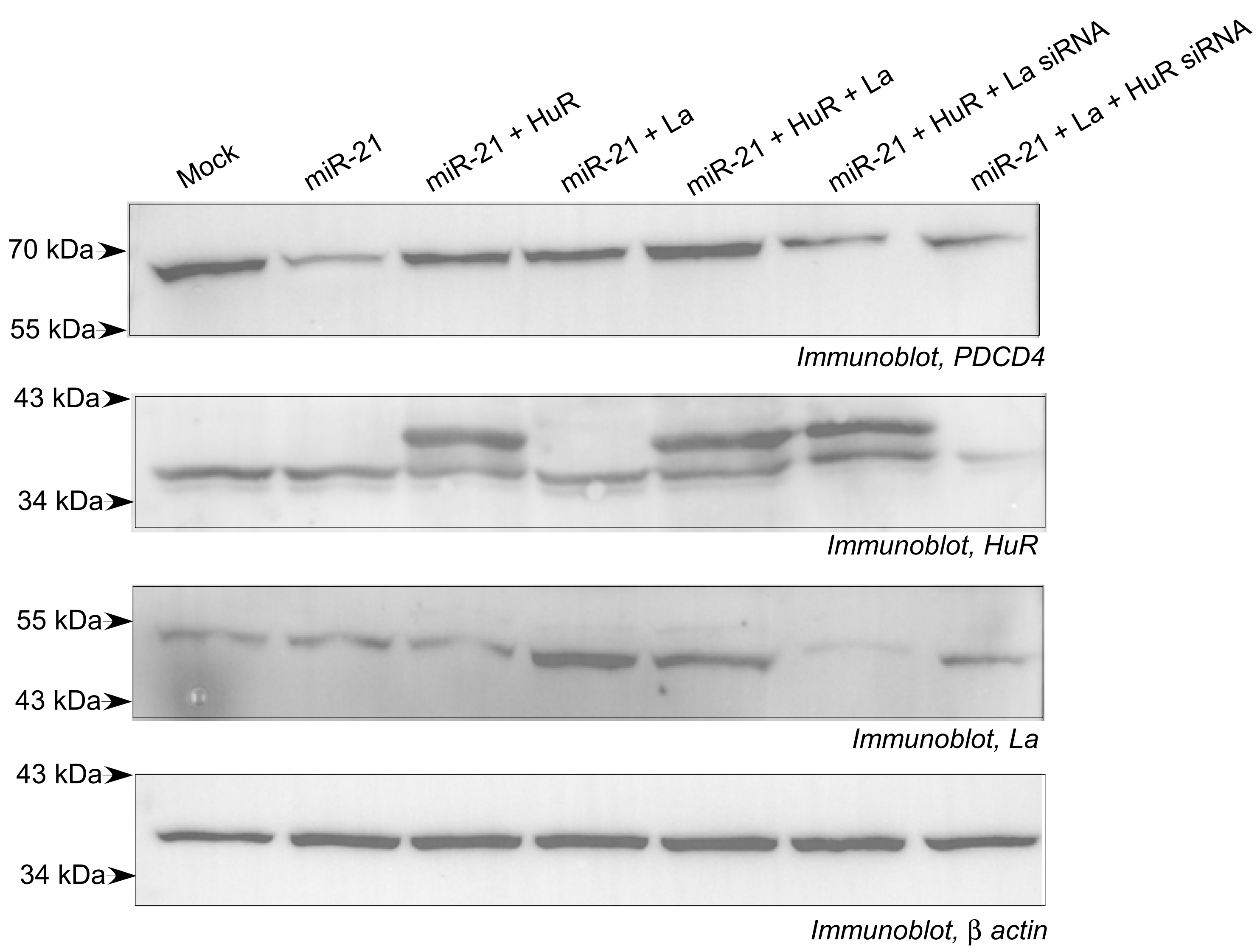

Fig S8. Immunoblot of cell lysates transfected with pSUPER-miR21 and either with pCDNA3-La (2 μg) or pCDNA3-HuR (2 μg) or both (1 μg each), or pCDNA3-HuR and La siRNA (100 pmole) or pCDNA3-La and HuR siRNA (100 pmole) using anti-PDCD4, anti-HuR, anti-La and anti-b-actin antibodies. The two bands in anti-HuR blot indicate myc-tagged and endogenous HuR protein.

Fig S9

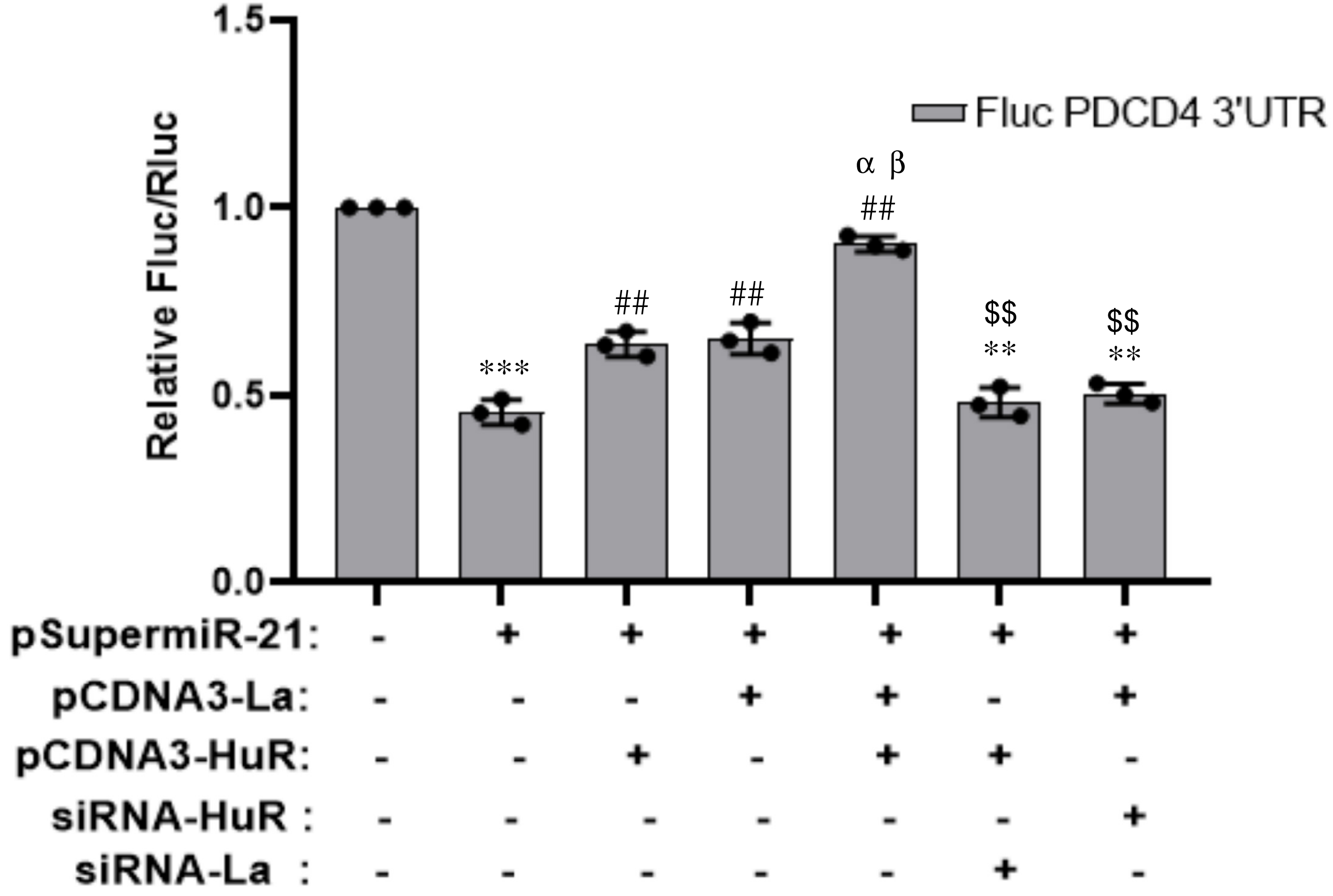

Fig S9. Luciferase reporter assay of MCF7 cells co-transfected with FlucPDCD4 3'UTR reporter gene construct together with pSUPER miR-21 and either pCDNA3-La (400 ng) or pCDNA3-HuR (400 ng) or both (200 ng each) or pCDNA3-HuR and La siRNA (10 pmole) or pCDNA3-La and HuR siRNA (10 pmole). Fluc values were normalised to Rluc as transfection control. ** and *** represents significant difference (p </= 0.01 and </= 0.005 respectively) from untransfected controls. ## represents significant difference (p </= 0.01) from miR-21 expression, α and β represents significant difference (p </= 0.05) with cells expressing HuR and La respectively. $$ represents significant difference (p </= 0.01) with cells expressing both HuR and La.

Fig S10

PDCD4 3'UTR (1-249) HuR-binding site mut

ATATAAGAACTCTTGCAGTCTTAGATGTTATAAAAATATATATCTGAATTGTAAGAG
TTGTTAGCACAAGTTTTTTTTTTTTTTTTTTTTAAGCACTTGTTTTGGGTACAAGG
CATTTCTGGGTTCAGTCTGTTAGGCCTCTGTGGGGAATTTTTAAAG
GAAATGTTTTTTCTTTTTTTTTTGTTTTTCGAGGGGGCAAGGAGGGACAGAAAA
GTAACCTCTTCTTAAGTGGAATATTCTAATAAGCTA

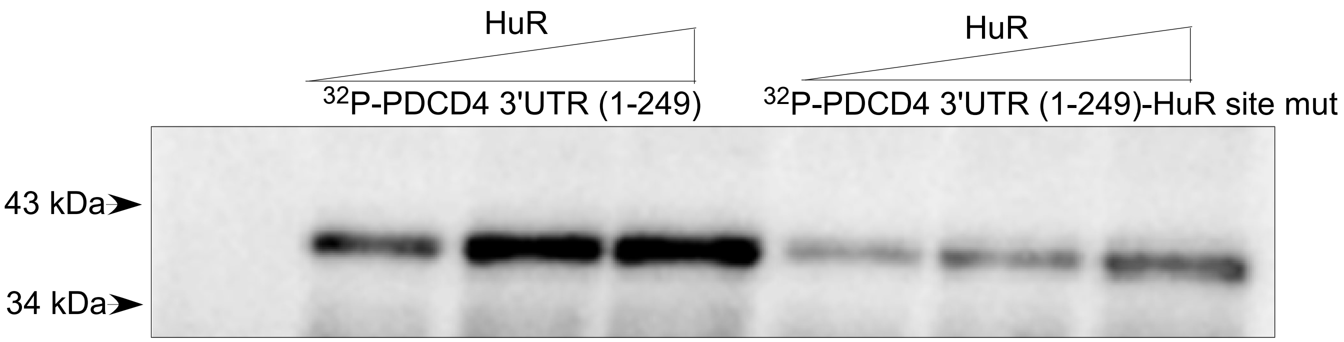

Fig. S10. Mutated sequence of HuR binding site in PDCD4 3’UTR RNA (1-249 nt) in red. *in vitro* binding of ^{32}P-labelled PDCD4 3'UTR-WT and HuR binding site mutant RNAs with purified HuR protein (10, 20 and 40 pmole).

Fig S11

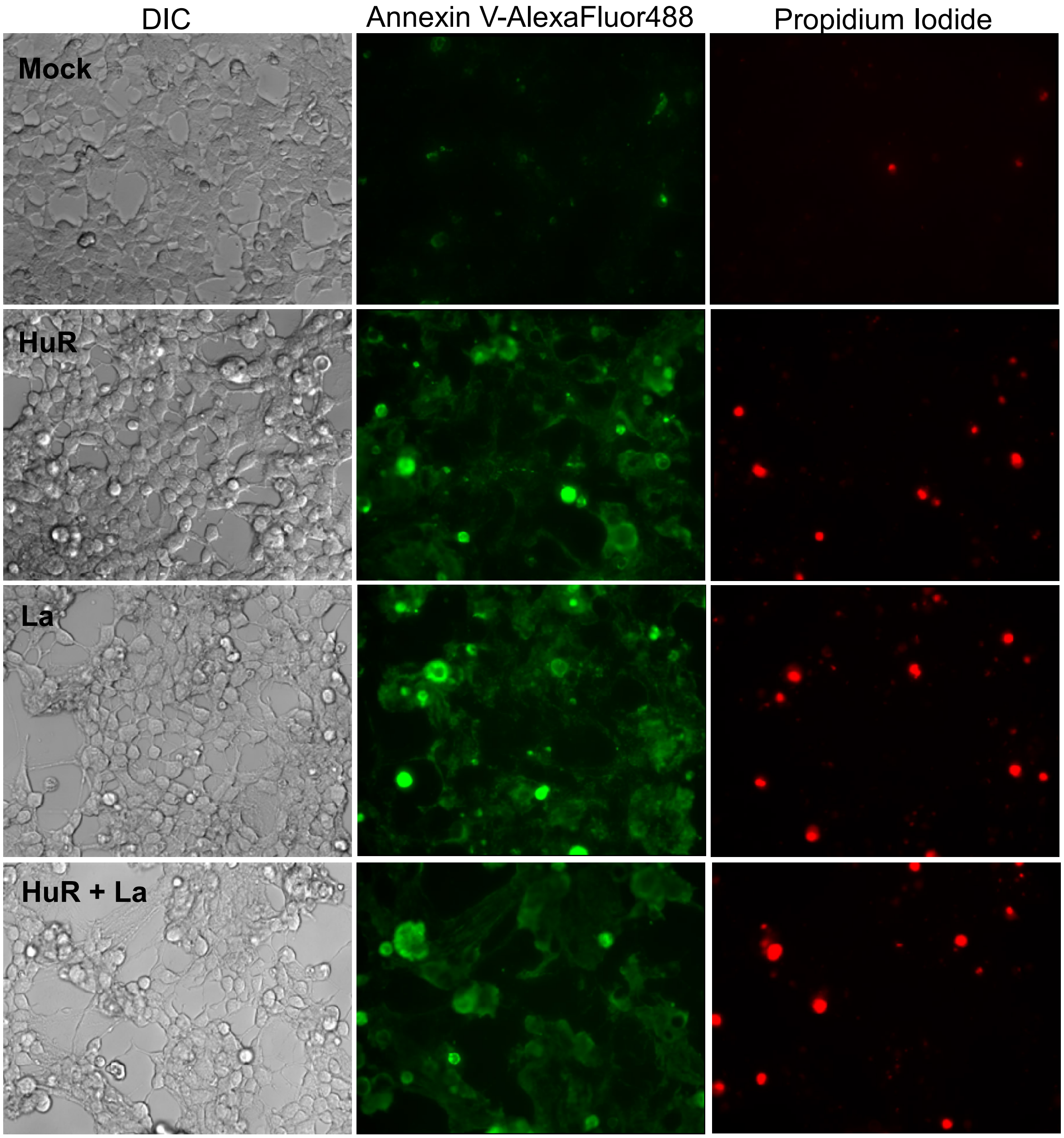

Fig. S11. MCF7 cells were mock transfected or transfected with La and HuR expressing constructs, individually (2 μg) or in combination (1 μg each). Cells were serum starved for 48 h to induce apoptosis and stained with AnexinV-AlexaFluor488 and PI and observed by fluorescent microscopy.

Fig S12

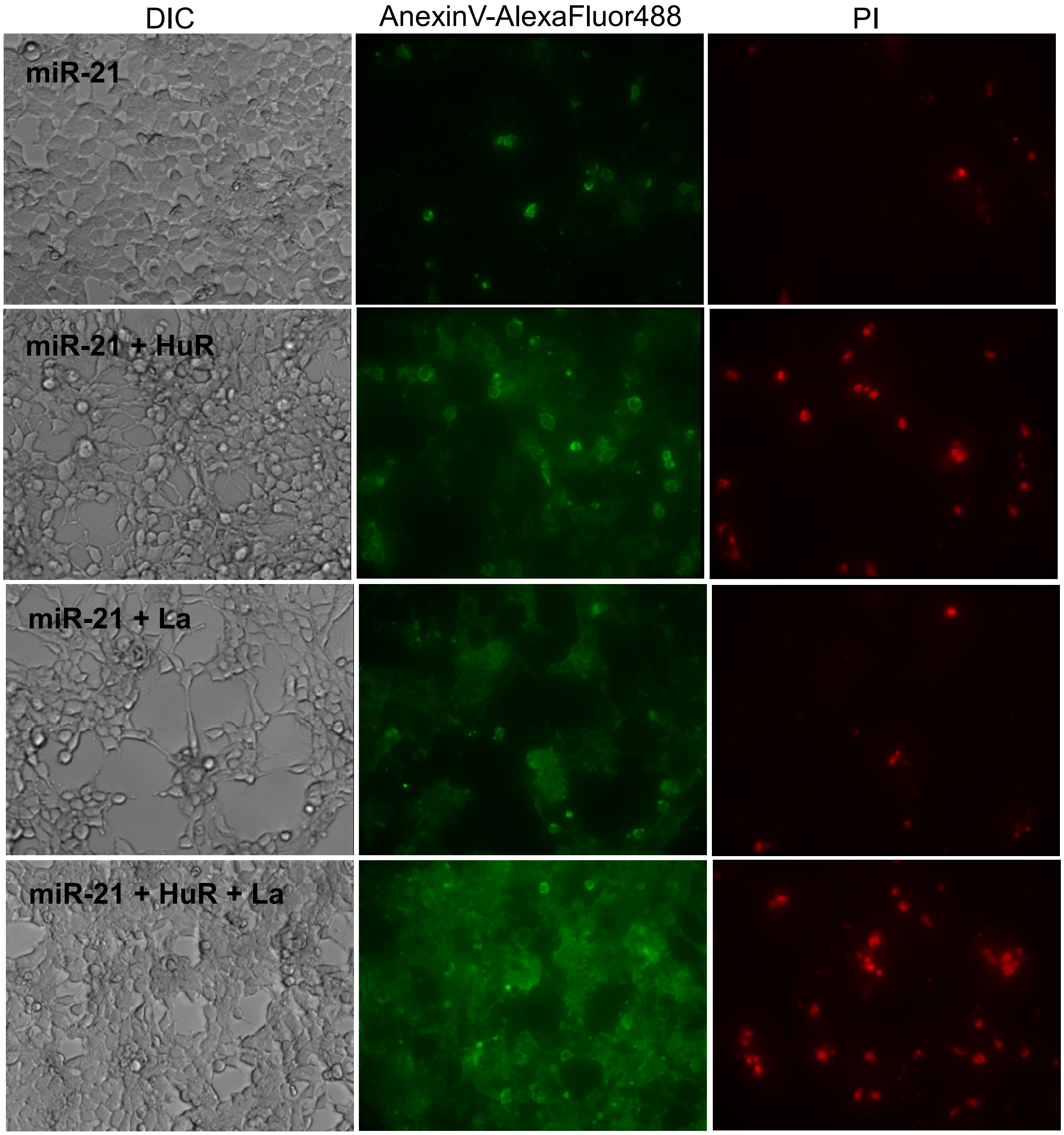

Fig. S12. MCF7 cells were transfected with a miR-21-expressing construct (2 μg) together with/without La and HuR expressing constructs, individually (2 μg) or in combination (1 μg each). Cells were serum starved for 48 h to induce apoptosis and stained with AnexinV-AlexaFluor488 and PI and observed by fluorescent microscopy.

Table S1. Partial List of proteins identified in mass spectrometric analysis of protein band indicated in Fig. 1A. The list has been filtered to remove common contaminants (keratin, trypsin), high molecular weight proteins (> 60 kDa), very low molecular weight proteins (<16 kDa), common structural proteins, and proteins identified by single peptides. Proteins with a Sum PEP score >2.00 has been shown. The Lupus La protein is highlighted.

Accession	Description	Sum PEP Score	Coverage	# Peptides	MW [kDa]
P00558	Phosphoglycerate kinase 1 OS=Homo sapiens OX=9606 GN=PGK1 PE=1 SV=3	20.221	27.57794	9	44.6
P68104	Elongation factor 1-alpha 1 OS=Homo sapiens OX=9606 GN=EEF1A1 PE=1 SV=1	11.306	18.83117	7	50.1
P04075-2	Isoform 2 of Fructose-bisphosphate aldolase A OS=Homo sapiens OX=9606 GN=ALDOA	9.118	16.74641	6	45.2
P12277	Creatine kinase B-type OS=Homo sapiens OX=9606 GN=CKB PE=1 SV=1	9.028	14.69816	4	42.6
P60842	Eukaryotic initiation factor 4A-I OS=Homo sapiens OX=9606 GN=EIF4A1 PE=1 SV=1	8.753	16.99507	6	46.1
P67809	Nuclease-sensitive element-binding protein 1 OS=Homo sapiens OX=9606 GN=YBX1 PE=1 SV=3	8.03	17.59259	3	35.9
P23526	Adenosylhomocysteinase OS=Homo sapiens OX=9606 GN=AHCY PE=1 SV=4	7.475	14.35185	6	47.7
P68371	Tubulin beta-4B chain OS=Homo sapiens OX=9606 GN=TUBB4B PE=1 SV=1	7.33	12.35955	5	49.8
A0A2U3TZH3	Elongation factor 1-alpha 2 OS=Homo sapiens OX=9606 GN=EEF1A2 PE=4 SV=1	7.246	12.70161	6	54.3
Q9UQ80	Proliferation-associated protein 2G4 OS=Homo sapiens OX=9606 GN=PA2G4 PE=1 SV=3	6.922	10.6599	4	43.8
P31153	S-adenosylmethionine synthase isoform type-2 OS=Homo sapiens OX=9606 GN=MAT2A PE=1 SV=1	6.338	8.607595	4	43.6
Q9Y266	Nuclear migration protein nudC OS=Homo sapiens OX=9606 GN=NUDC PE=1 SV=1	6.146	11.17825	4	38.2
P07355-2	Isoform 2 of Annexin A2 OS=Homo sapiens OX=9606 GN=ANXA2	5.544	11.20448	4	40.4
P38919	Eukaryotic initiation factor 4A-III OS=Homo sapiens OX=9606 GN=EIF4A3 PE=1 SV=4	5.439	11.67883	4	46.8
J3KQ32	Obg-like ATPase 1 OS=Homo sapiens OX=9606 GN=OLA1 PE=1 SV=1	5.077	9.134615	3	46.9
P50395	Rab GDP dissociation inhibitor beta OS=Homo sapiens OX=9606 GN=GDI2 PE=1 SV=2	4.942	9.88764	3	50.6
P52209	6-phosphogluconate dehydrogenase, decarboxylating OS=Homo sapiens OX=9606 GN=PGD PE=1 SV=3	4.268	6.625259	3	53.1
A0A2R8Y6G6	Alpha-enolase OS=Homo sapiens OX=9606 GN=ENO1 PE=1 SV=1	4.172	5.529954	3	47.3
O75821	Eukaryotic translation initiation factor 3 subunit G OS=Homo sapiens OX=9606 GN=EIF3G PE=1 SV=2	4.066	14.0625	3	35.6
Q15008-4	Isoform 4 of 26S proteasome non-ATPase regulatory subunit 6 OS=Homo sapiens OX=9606 GN=PSMD6	3.144	3.846154	2	51.9
Q9UNZ2-5	Isoform 3 of NSFL1 cofactor p47 OS=Homo sapiens OX=9606 GN=NSFL1C	3.111	5.913978	2	40.8
P49411	Elongation factor Tu, mitochondrial OS=Homo sapiens OX=9606 GN=TUFM PE=1 SV=2	2.9	5.752212	2	49.5
Q12905	Interleukin enhancer-binding factor 2 OS=Homo sapiens OX=9606 GN=ILF2 PE=1 SV=2	2.673	7.179487	2	43
P22626	Heterogeneous nuclear ribonucleoproteins A2/B1 OS=Homo sapiens OX=9606 GN=HNRNPA2B1 PE=1 SV=2	2.523	5.665722	2	37.4
P08670	Vimentin OS=Homo sapiens OX=9606 GN=VIM PE=1 SV=4	2.475	3.648069	2	53.6
Q15293	Reticulocalbin-1 OS=Homo sapiens OX=9606 GN=RCN1 PE=1 SV=1	2.416	6.042296	2	38.9
P05455	Lupus La protein OS=Homo sapiens OX=9606 GN=SSB PE=1 SV=2	2.371	5.147059	2	46.8
Q7L1Q6-3	Isoform 3 of Basic leucine zipper and W2 domain-containing protein 1 OS=Homo sapiens OX=9606 GN=BZW1	2.223	4.21286	2	51.2
Q13838-2	Isoform 2 of Spliceosome RNA helicase DDX39B OS=Homo sapiens OX=9606 GN=DDX39B	2.154	3.386005	2	50.6

Mass spectrometry search parameters:

Name of peaklist-generating software and release version (number or date): Proteome Discoverer version 2.1.1.21

Name of the search engine and release version (number or date): SequestHT

Name of sequence database searched and release version/date: Human protein database downloaded from Uniprot on 2019

Number of entries in the database actually searched: Entries in the Human protein database downloaded - 42,300

Specificity of all proteases used to generate peptides: Trypsin

Number of missed and/or non-specific cleavages permitted: 2 mis-cleavages

List of all fixed & variable modifications (including residue specificity) considered:
Fixed modifications - Carbamidomethyl (C)
Variable modifications - Oxidation (M), Acetyl at Protein N term

Mass tolerance for precursor ions: 10 ppm

Mass tolerance for fragment ions: 0.6 Da

Threshold score/Expectation value for accepting individual spectra: Based on FDR

Estimation of false discovery rate (FDR) and how calculated (for large datasets): FDR - 0.01 (1%)

Kinetics: ‘HuR’, fit: ‘1. 1:1 Binding’

Curve: Fc=2-1 Ligand: N/A Sample: HuR Temp: 25°C

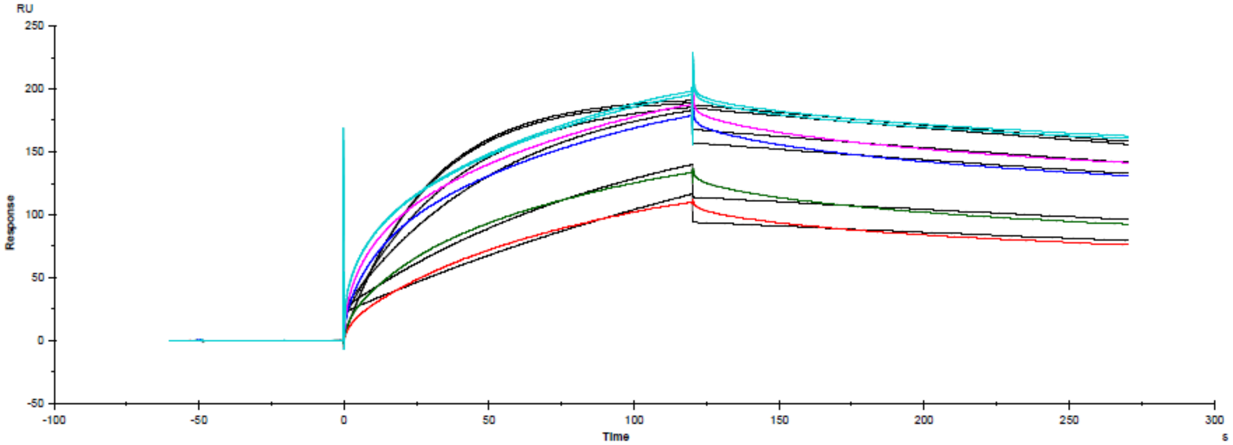

Residuals

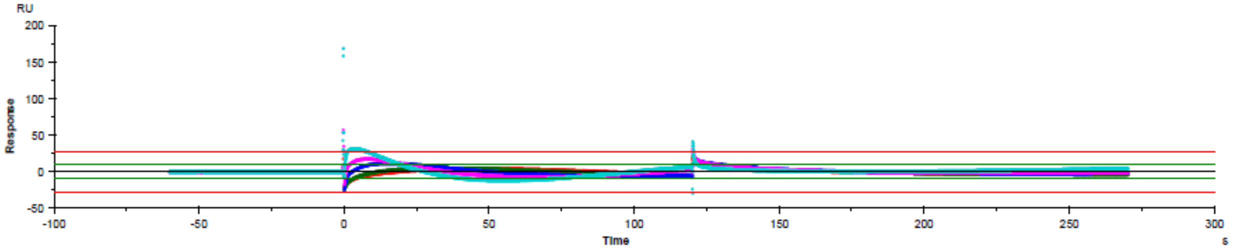

Kinetics: ‘HuR’, fit: ‘1. 1:1 Binding’ (continued)

Report table

Curve	ka (1/Ms)	kd (1/s)	KD (M)	Rmax (RU)	Conc (M)	tc	Flow (ul/min)	kt (RU/Ms)	RI (RU)	Chi² (RU²)	U-value
	3.354E+7	0.001131	3.373E-11			6.044E+19				35.5	4
Cycle: 25 0.1 nM				301.8	1.000E-10		30.00	1.878E+20	22.31		
Cycle: 26 0.25 nM				189.7	2.500E-10		30.00	1.878E+20	25.91		
Cycle: 27 0.5 nM				189.7	5.000E-10		30.00	1.878E+20	25.83		
Cycle: 28 0.75 nM				182.8	7.500E-10		30.00	1.878E+20	17.76		
Cycle: 29 1 nM				196.6	1.000E-9		30.00	1.878E+20	3.412		
Cycle: 30 1 nM				193.8	1.000E-9		30.00	1.878E+20	3.879		

Parameters table

Curve	ka (1/Ms)	SE(ka)	kd (1/s)	SE(kd)	Rmax (RU)	SE(Rmax)	Conc (M)	tc	SE(tc)	f (ul/min)	RI (RU)	SE(RI)
	3.354E+7	1.7E+5	0.001131	9.5E-6				6.044E+19	4.6E+22			
Cycle: 25 0.1 nM					301.8	1.4	1E-10			30	22.3	0.20
Cycle: 26 0.25 nM					189.7	0.65	2.5E-10			30	25.9	0.21
Cycle: 27 0.5 nM					189.7	0.41	5E-10			30	25.8	0.25
Cycle: 28 0.75 nM					182.8	0.29	7.5E-10			30	17.8	0.27
Cycle: 29 1 nM					196.6	0.26	1E-09			30	3.4	0.29
Cycle: 30 1 nM					193.8	0.25	1E-09			30	3.9	0.29

Kinetics: 'HuR', fit: '1. 1:1 Binding'

Curve: Fc=4-3 Ligand: N/A Sample: HuR Temp: 25°C

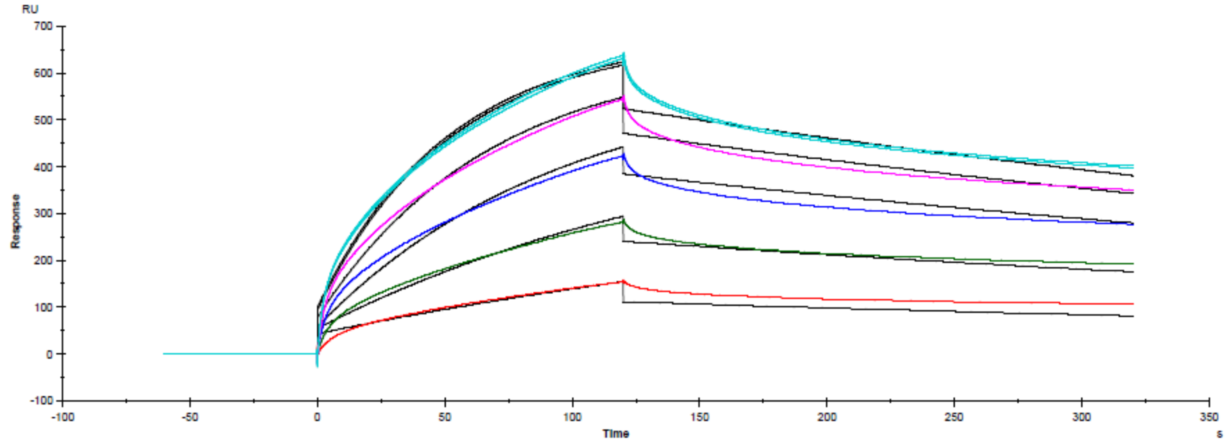

Residuals

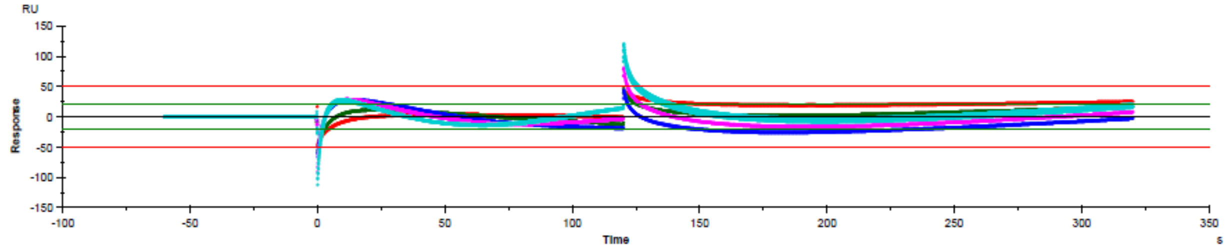

Kinetics: 'HuR', fit: '1. 1:1 Binding' (continued)

Report table

Curve	ka (1/Ms)	kd (1/s)	KD (M)	Rmax (RU)	Conc (M)	tc	Flow (ul/min)	kt (RU/Ms)	RI (RU)	Chi² (RU²)	U-value
	1.784E+4	0.001590	8.909E-8	631.5		2.499E+16				205	2
Cycle: 16 0.1 µM					1.000E-7		30.00	7.765E+16	43.07		
Cycle: 17 0.25 µM					2.500E-7		30.00	7.765E+16	53.87		
Cycle: 18 0.5 µM					5.000E-7		30.00	7.765E+16	57.85		
Cycle: 19 0.75 µM					7.500E-7		30.00	7.765E+16	77.41		
Cycle: 20 1 µM					1.000E-6		30.00	7.765E+16	99.52		
Cycle: 21 1 µM					1.000E-6		30.00	7.765E+16	93.06		

Parameters table

Curve	ka (1/Ms)	SE(ka)	kd (1/s)	SE(kd)	Rmax (RU)	SE(Rmax)	Conc (M)	tc	SE(tc)	f (ul/min)	RI (RU)	SE(RI)
	1.784E+4	43	0.001590	6.1E-6	631.5	0.79		2.499E+16	8.6E+18			
Cycle: 16 0.1 µM							1E-07			30	43.1	0.42
Cycle: 17 0.25 µM							2.5E-07			30	53.9	0.45
Cycle: 18 0.5 µM							5E-07			30	57.9	0.48
Cycle: 19 0.75 µM							7.5E-07			30	77.4	0.49
Cycle: 20 1 µM							1E-06			30	99.5	0.50
Cycle: 21 1 µM							1E-06			30	93.1	0.50

Kinetics: 'HuR', fit: '1. 1:1 Binding'

Curve: Fc=2-1 Ligand: mRNA_PDCD4-UTR Sample: HuR Temp: 25°C

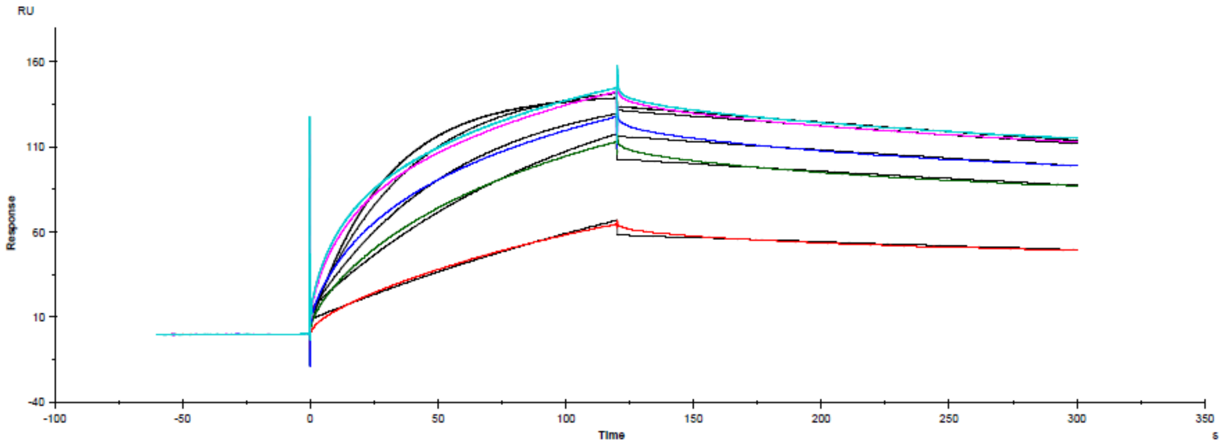

Residuals

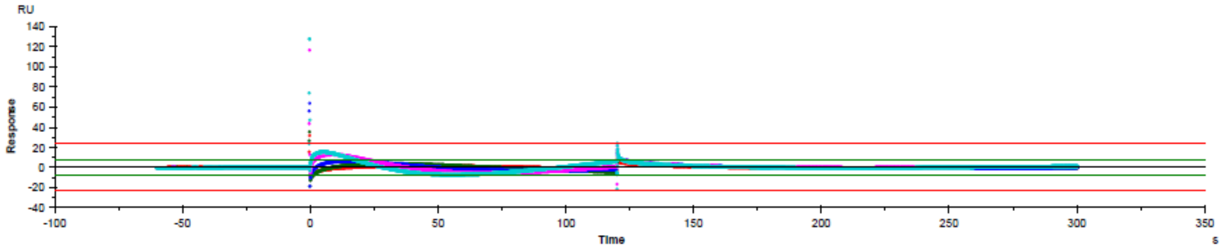

Kinetics: 'HuR', fit: '1. 1:1 Binding' (continued)

Report table

Curve	ka (1/Ms)	kd (1/s)	KD (M)	Rmax (RU)	Conc (M)	tc	Flow (ul/min)	kt (RU/Ms)	RI (RU)	Chi² (RU²)	U-value
	3.350E+4	9.015E-4	2.691E-8			2.118E+17				12.8	3
Cycle: 19 0.1 µM				185.0	1.000E-7		30.00	6.582E+17	8.654		
Cycle: 20 0.25 µM				169.7	2.500E-7		30.00	6.582E+17	14.80		
Cycle: 21 0.5 µM				139.3	5.000E-7		30.00	6.582E+17	13.21		
Cycle: 22 0.75 µM				142.5	7.500E-7		30.00	6.582E+17	9.969		
Cycle: 23 1 µM				139.7	1.000E-6		30.00	6.582E+17	4.952		
Cycle: 24 1 µM				139.5	1.000E-6		30.00	6.582E+17	4.844		

Parameters table

Curve	ka (1/Ms)	SE(ka)	kd (1/s)	SE(kd)	Rmax (RU)	SE(Rmax)	Conc (M)	tc	SE(tc)	f (ul/min)	RI (RU)	SE(RI)
	3.350E+4	1.4E+2	9.015E-4	5.8E-6				2.118E+17	3.4E+20			
Cycle: 19 0.1 µM					185.0	0.69	1E-07			30	8.7	0.12
Cycle: 20 0.25 µM					169.7	0.45	2.5E-07			30	14.8	0.13
Cycle: 21 0.5 µM					139.3	0.23	5E-07			30	13.2	0.14
Cycle: 22 0.75 µM					142.5	0.16	7.5E-07			30	10.0	0.16
Cycle: 23 1 µM					139.7	0.13	1E-06			30	5.0	0.17
Cycle: 24 1 µM					139.5	0.13	1E-06			30	4.8	0.17

Kinetics: ‘La’, fit: ‘1. 1:1 Binding’

Curve: Fc=2-1 Ligand: N/A Sample: La Temp: 25°C

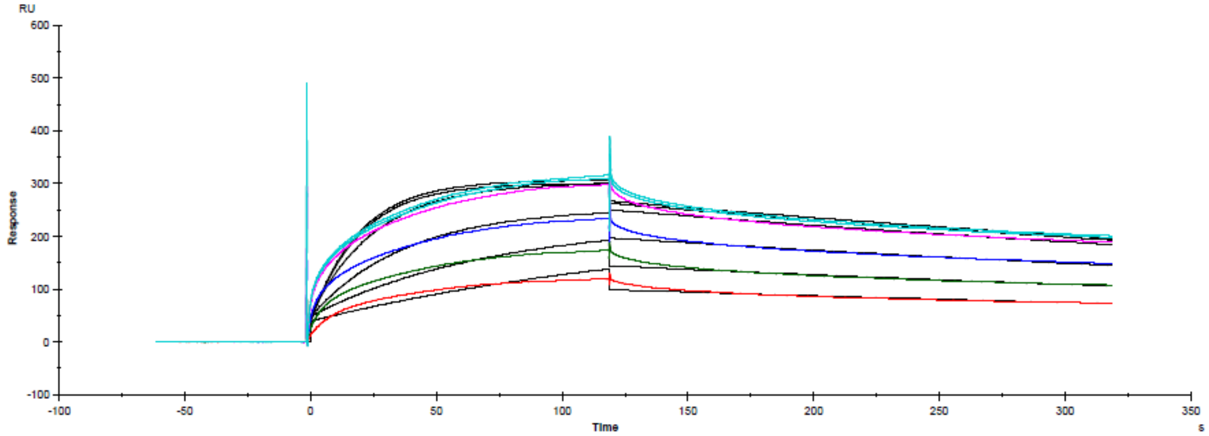

Residuals

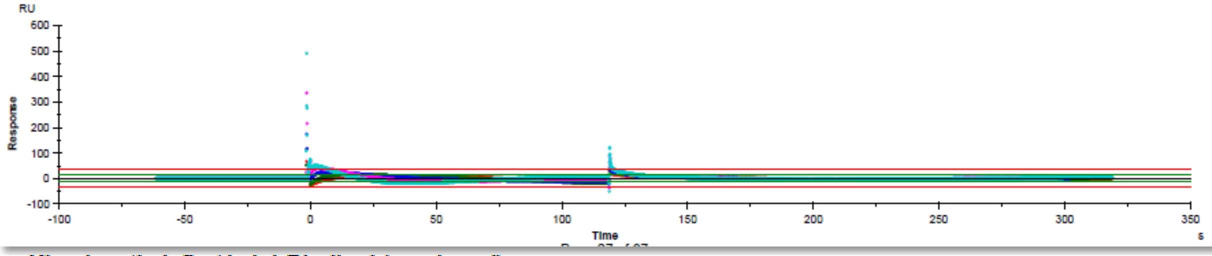

Kinetics: ‘La’, fit: ‘1. 1:1 Binding’ (continued)

Report table

Curve	ka (1/Ms)	kd (1/s)	KD (M)	Rmax (RU)	Conc (M)	tc	Flow (ul/min)	kt (RU/Ms)	RI (RU)	Chi² (RU²)	U-value
	4.983E+4	0.001541	3.093E-8			3.177E+21				114	3
Cycle: 7 0.1 µM				240.6	1.000E-7		30.00	9.872E+21	39.39		
Cycle: 8 0.25 µM				200.1	2.500E-7		30.00	9.872E+21	48.40		
Cycle: 9 0.5 µM				218.8	5.000E-7		30.00	9.872E+21	47.64		
Cycle: 10 0.75 µM				263.4	7.500E-7		30.00	9.872E+21	49.09		
Cycle: 11 1 µM				275.8	1.000E-6		30.00	9.872E+21	39.01		
Cycle: 12 1 µM				271.2	1.000E-6		30.00	9.872E+21	37.42		

Parameters table

Curve	ka (1/Ms)	SE(ka)	kd (1/s)	SE(kd)	Rmax (RU)	SE(Rmax)	Conc (M)	tc	SE(tc)	f (ul/min)	RI (RU)	SE(RI)
	4.983E+4	2.8E+2	0.001541	8.8E-6				3.177E+21	1.2E+26			
Cycle: 7 0.1 µM					240.6	1.2	1E-07			30	39.4	0.35
Cycle: 8 0.25 µM					200.1	0.65	2.5E-07			30	48.4	0.37
Cycle: 9 0.5 µM					218.8	0.42	5E-07			30	47.6	0.43
Cycle: 10 0.75 µM					263.4	0.39	7.5E-07			30	49.1	0.48
Cycle: 11 1 µM					275.8	0.38	1E-06			30	39.0	0.49
Cycle: 12 1 µM					271.2	0.38	1E-06			30	37.4	0.49

Kinetics: 'La', fit: '1. 1:1 Binding'

Curve: Fc=2-1 Ligand: N/A Sample: La Temp: 25°C

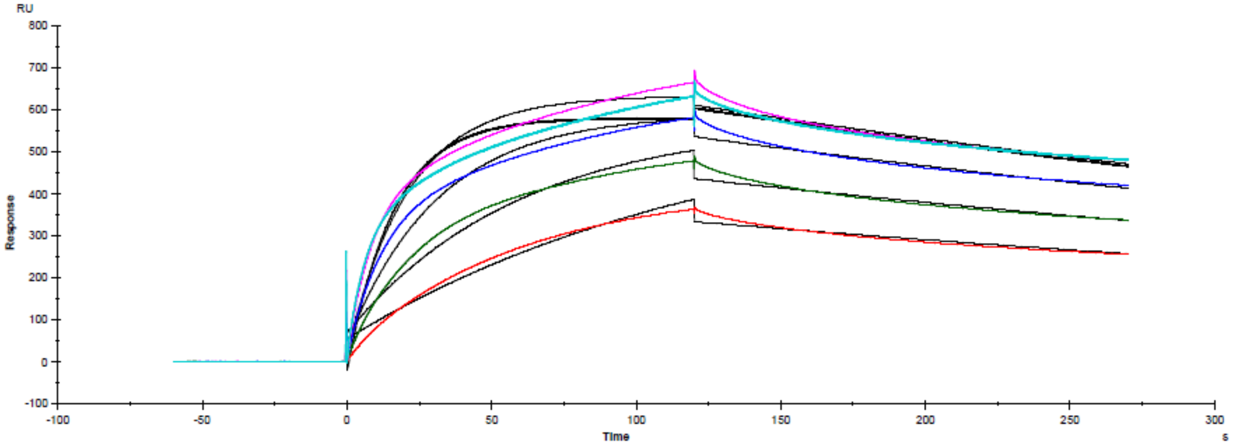

Residuals

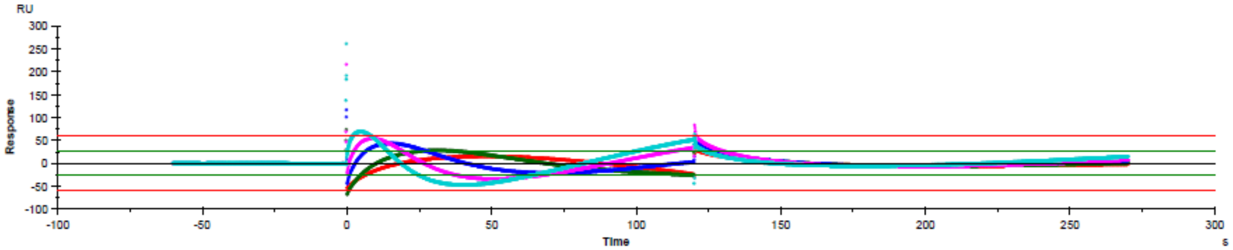

Kinetics: 'La', fit: '1. 1:1 Binding' (continued)

Report table

Curve	ka (1/Ms)	kd (1/s)	KD (M)	Rmax (RU)	Conc (M)	tc	Flow (ul/min)	kt (RU/Ms)	RI (RU)	Chi² (RU²)	U-value
	6.028E+7	0.001741	2.888E-11			2.311E+19				321	2
Cycle: 7 0.1 nM				707.3	1.000E-10		30.00	7.180E+19	53.32		
Cycle: 8 0.25 nM				560.2	2.500E-10		30.00	7.180E+19	67.51		
Cycle: 9 0.5 nM				578.5	5.000E-10		30.00	7.180E+19	42.21		
Cycle: 10 0.75 nM				636.1	7.500E-10		30.00	7.180E+19	19.40		
Cycle: 11 1 nM				622.6	1.000E-9		30.00	7.180E+19	-24.01		
Cycle: 12 1 nM				619.5	1.000E-9		30.00	7.180E+19	-24.16		

Parameters table

Curve	ka (1/Ms)	SE(ka)	kd (1/s)	SE(kd)	Rmax (RU)	SE(Rmax)	Conc (M)	tc	SE(tc)	f (ul/min)	RI (RU)	SE(RI)
	6.028E+7	2.2E+5	0.001741	8.9E-6				2.311E+19	4.0E+21			
Cycle: 7 0.1 nM					707.3	2.2	1E-10			30	53.3	0.61
Cycle: 8 0.25 nM					560.2	1.1	2.5E-10			30	67.5	0.68
Cycle: 9 0.5 nM					578.5	0.76	5E-10			30	42.2	0.79
Cycle: 10 0.75 nM					636.1	0.72	7.5E-10			30	19.4	0.84
Cycle: 11 1 nM					622.6	0.69	1E-09			30	-24.0	0.83
Cycle: 12 1 nM					619.5	0.69	1E-09			30	-24.2	0.82

Kinetics: 'La', fit: '1. 1:1 Binding'

Curve: Fc=2-1 Ligand: mRNA_PDCD4-UTR Sample: La Temp: 25°C

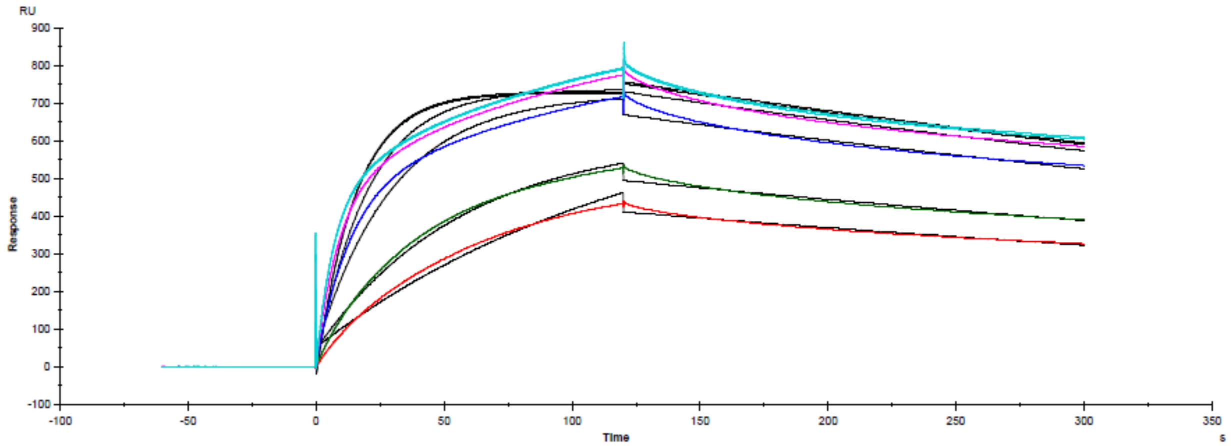

Residuals

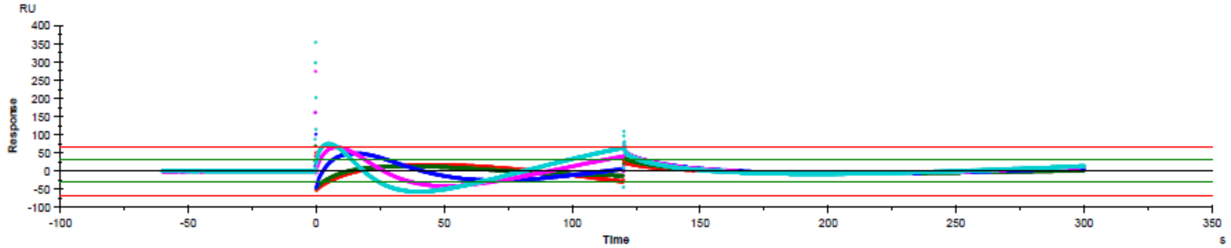

Kinetics: 'La', fit: '1. 1:1 Binding' (continued)

Report table

Curve	ka (1/Ms)	kd (1/s)	KD (M)	Rmax (RU)	Conc (M)	tc	Flow (ul/min)	kt (RU/Ms)	RI (RU)	Chi² (RU²)	U-value
	6.469E+4	0.001344	2.078E-8			1.233E+18				377	2
Cycle: 7 0.1 µM				815.9	1.000E-7		30.00	3.831E+18	51.26		
Cycle: 8 0.25 µM				610.4	2.500E-7		30.00	3.831E+18	45.89		
Cycle: 9 0.5 µM				709.5	5.000E-7		30.00	3.831E+18	42.43		
Cycle: 10 0.75 µM				752.3	7.500E-7		30.00	3.831E+18	4.715		
Cycle: 11 1 µM				773.0	1.000E-6		30.00	3.831E+18	-26.92		
Cycle: 12 1 µM				767.6	1.000E-6		30.00	3.831E+18	-25.37		

Parameters table

Curve	ka (1/Ms)	SE(ka)	kd (1/s)	SE(kd)	Rmax (RU)	SE(Rmax)	Conc (M)	tc	SE(tc)	f (ul/min)	RI (RU)	SE(RI)
	6.469E+4	2.1E+2	0.001344	6.0E-6				1.233E+18	1.4E+21			
Cycle: 7 0.1 µM					815.9	2.1	1E-07			30	51.3	0.65
Cycle: 8 0.25 µM					610.4	0.98	2.5E-07			30	45.9	0.71
Cycle: 9 0.5 µM					709.5	0.72	5E-07			30	42.4	0.83
Cycle: 10 0.75 µM					752.3	0.68	7.5E-07			30	4.7	0.86
Cycle: 11 1 µM					773.0	0.67	1E-06			30	-26.9	0.86
Cycle: 12 1 µM					767.6	0.67	1E-06			30	-25.4	0.86

Kinetics: ‘HuR_La’, fit: ‘1. 1:1 Binding’

Curve: Fc=2-1 Ligand: N/A Sample: HuR_La Temp: 25°C

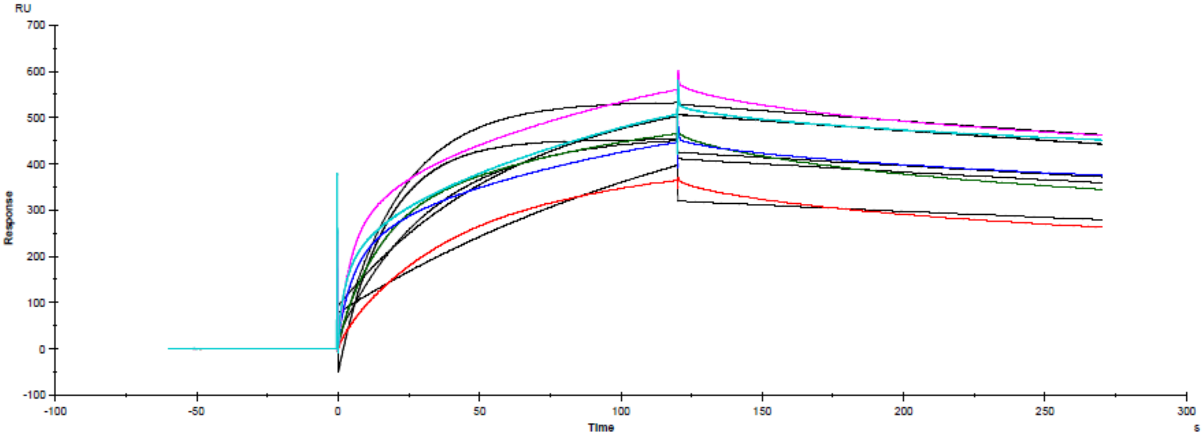

Residuals

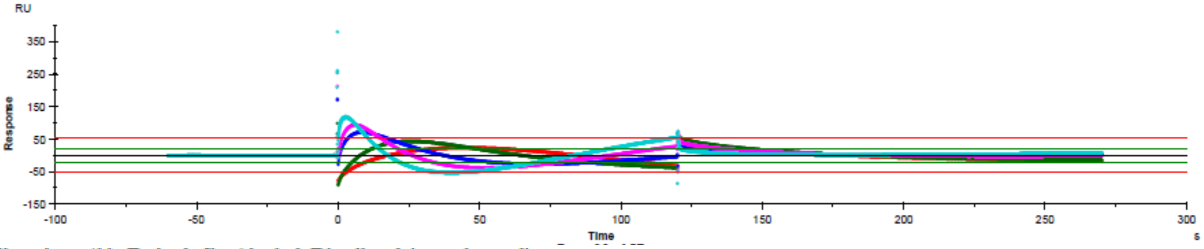

Kinetics: ‘HuR_La’, fit: ‘1. 1:1 Binding’ (continued)

Report table

Curve	ka (1/Ms)	kd (1/s)	KD (M)	Rmax (RU)	Conc (M)	tc	Flow (ul/min)	kt (RU/Ms)	RI (RU)	Chi² (RU²)	U-value
	5.865E+7	8.956E-4	1.527E-11			1.312E+17				534	7
Cycle: 16 0.1 nM				662.0	1.000E-10		30.00	4.078E+17	77.38		
Cycle: 17 0.25 nM				515.3	2.500E-10		30.00	4.078E+17	92.05		
Cycle: 18 0.5 nM				449.9	5.000E-10		30.00	4.078E+17	25.37		
Cycle: 19 0.75 nM				542.2	7.500E-10		30.00	4.078E+17	3.506		
Cycle: 20 1 nM				514.3	1.000E-9		30.00	4.078E+17	-53.70		
Cycle: 21 1 nM				514.9	1.000E-9		30.00	4.078E+17	-54.39		

Parameters table

Curve	ka (1/Ms)	SE(ka)	kd (1/s)	SE(kd)	Rmax (RU)	SE(Rmax)	Conc (M)	tc	SE(tc)	f (ul/min)	RI (RU)	SE(RI)
	5.865E+7	3.3E+5	8.956E-4	1.2E-5				1.312E+17	2.1E+18			
Cycle: 16 0.1 nM					662.0	3.0	1E-10			30	77.4	0.78
Cycle: 17 0.25 nM					515.3	1.5	2.5E-10			30	92.1	0.88
Cycle: 18 0.5 nM					449.9	0.88	5E-10			30	25.4	0.98
Cycle: 19 0.75 nM					542.2	0.88	7.5E-10			30	3.5	1.1
Cycle: 20 1 nM					514.3	0.84	1E-09			30	-53.7	1.0
Cycle: 21 1 nM					514.9	0.84	1E-09			30	-54.4	1.0

Kinetics: 'HuR_La 4', fit: '1. 1:1 Binding'

Curve: Fc=4-3 Ligand: N/A Sample: HuR_La Temp: 25°C

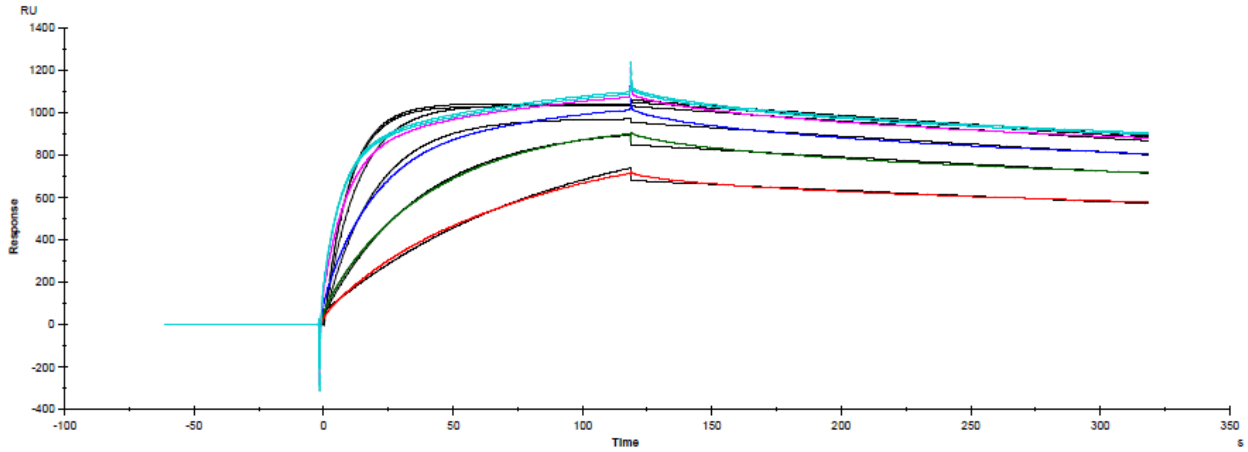

Residuals

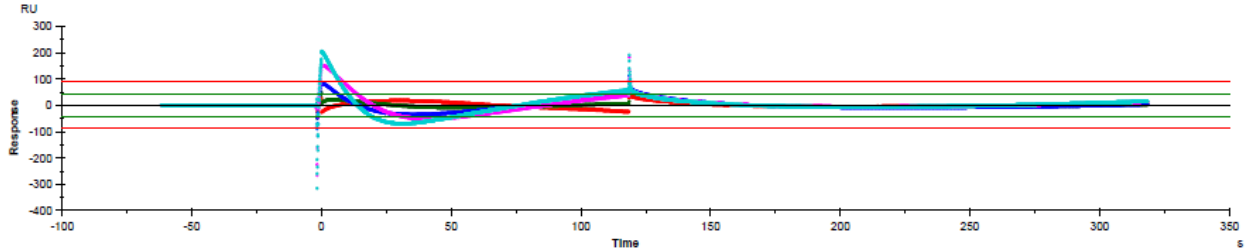

Kinetics: 'HuR_La 4', fit: '1. 1:1 Binding' (continued)

Report table

Curve	ka (1/Ms)	kd (1/s)	KD (M)	Rmax (RU)	Conc (M)	tc	Flow (ul/min)	kt (RU/Ms)	RI (RU)	Chi² (RU²)	U-value
	1.026E+5	8.712E-4	8.489E-9			1.283E+17				621	3
Cycle: 25 0.1 µM				1005	1.000E-7		30.00	3.985E+17	58.80		
Cycle: 26 0.25 µM				916.2	2.500E-7		30.00	3.985E+17	45.56		
Cycle: 27 0.5 µM				971.6	5.000E-7		30.00	3.985E+17	14.56		
Cycle: 28 0.75 µM				1042	7.500E-7		30.00	3.985E+17	4.777		
Cycle: 29 1 µM				1068	1.000E-6		30.00	3.985E+17	-17.61		
Cycle: 30 1 µM				1058	1.000E-6		30.00	3.985E+17	-19.81		

Parameters table

Curve	ka (1/Ms)	SE(ka)	kd (1/s)	SE(kd)	Rmax (RU)	SE(Rmax)	Conc (M)	tc	SE(tc)	f (ul/min)	RI (RU)	SE(RI)
	1.026E+5	2.9E+2	8.712E-4	4.4E-6				1.283E+17	3.1E+19			
Cycle: 25 0.1 µM					1004.7	1.8	1E-07			30	58.8	0.85
Cycle: 26 0.25 µM					916.2	0.89	2.5E-07			30	45.6	0.99
Cycle: 27 0.5 µM					971.6	0.76	5E-07			30	14.6	1.0
Cycle: 28 0.75 µM					1041.9	0.77	7.5E-07			30	4.8	1.0
Cycle: 29 1 µM					1067.9	0.77	1E-06			30	-17.6	1.0
Cycle: 30 1 µM					1058.3	0.77	1E-06			30	-19.8	1.0

Kinetics: 'HuR La 4', fit: '1. 1:1 Binding'

Curve: Fc=2-1 Ligand: mRNA_PDCD4-UTR Sample: HuR La Temp: 25°C

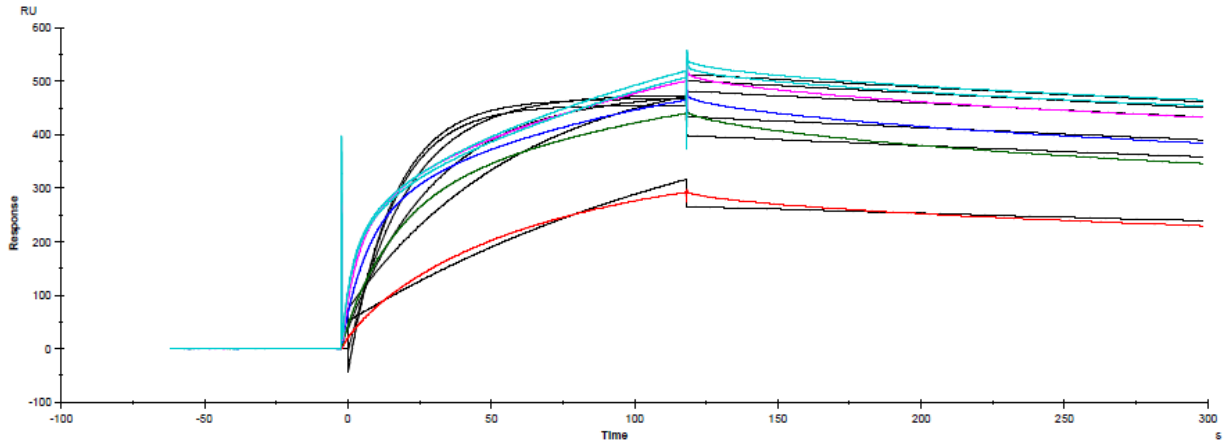

Residuals

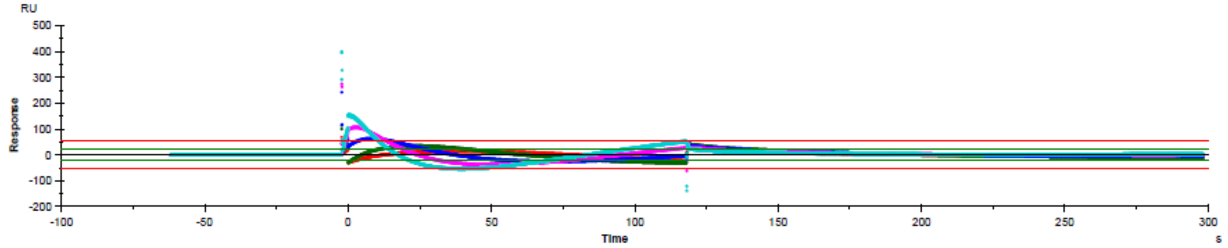

Kinetics: 'HuR La 4', fit: '1. 1:1 Binding' (continued)

Report table

Curve	ka (1/Ms)	kd (1/s)	KD (M)	Rmax (RU)	Conc (M)	tc	Flow (ul/min)	kt (RU/Ms)	RI (RU)	Chi² (RU²)	U-value
	6.271E+4	5.882E-4	9.380E-9			1.038E+21				512	9
Cycle: 31 0.1 µM				524.0	1.000E-7		30.00	3.225E+21	50.70		
Cycle: 32 0.25 µM				484.4	2.500E-7		30.00	3.225E+21	73.67		
Cycle: 33 0.5 µM				452.3	5.000E-7		30.00	3.225E+21	35.22		
Cycle: 34 0.75 µM				489.5	7.500E-7		30.00	3.225E+21	-8.124		
Cycle: 35 1 µM				518.2	1.000E-6		30.00	3.225E+21	-46.62		
Cycle: 36 1 µM				506.2	1.000E-6		30.00	3.225E+21	-45.91		

Parameters table

Curve	ka (1/Ms)	SE(ka)	kd (1/s)	SE(kd)	Rmax (RU)	SE(Rmax)	Conc (M)	tc	SE(tc)	f (ul/min)	RI (RU)	SE(RI)
	6.271E+4	3.5E+2	5.882E-4	9.6E-6				1.038E+21	8.4E+24			
Cycle: 31 0.1 µM					524.0	2.3	1E-07			30	50.7	0.75
Cycle: 32 0.25 µM					484.4	1.2	2.5E-07			30	73.7	0.85
Cycle: 33 0.5 µM					452.3	0.77	5E-07			30	35.2	0.95
Cycle: 34 0.75 µM					489.5	0.74	7.5E-07			30	-8.1	0.99
Cycle: 35 1 µM					518.2	0.74	1E-06			30	-46.6	0.99
Cycle: 36 1 µM					506.2	0.74	1E-06			30	-45.9	0.98

Kinetics: 'HuRMut_La1', fit: '1. 1:1 Binding'

Curve: Fc=2-1 Ligand: N/A Sample: HuRMut_La1 Temp: 25°C

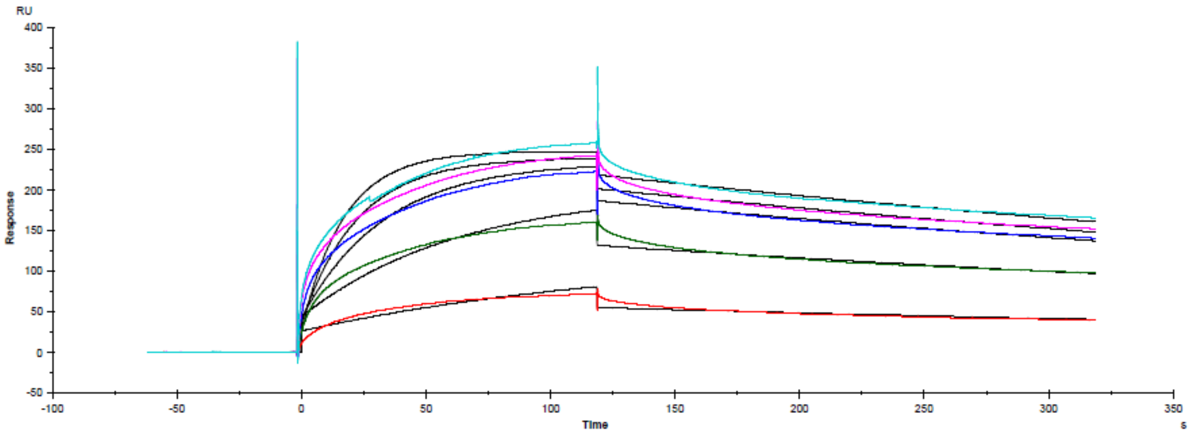

Residuals

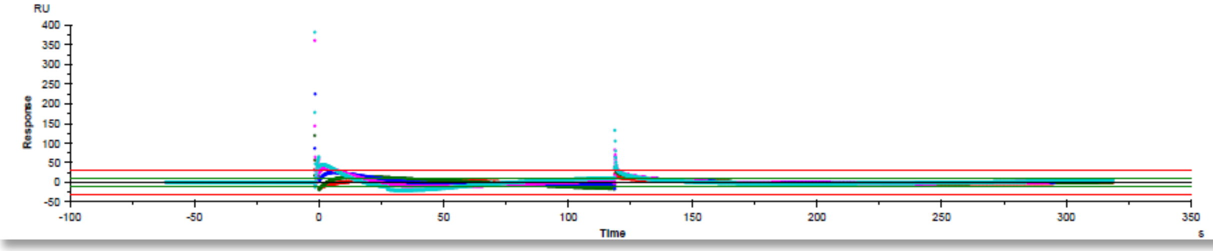

Kinetics: 'HuRMut_La1', fit: '1. 1:1 Binding' (continued)

Report table

Curve	ka (1/Ms)	kd (1/s)	KD (M)	Rmax (RU)	Conc (M)	tc	Flow (ul/min)	kt (RU/Ms)	RI (RU)	Chi² (RU²)	U-value
	5.678E+4	0.001542	2.715E-8			4.815E+17				78.2	3
Cycle: 17 0.1 µM				120.9	1.000E-7		30.00	1.496E+18	25.92		
Cycle: 18 0.25 µM				172.3	2.500E-7		30.00	1.496E+18	43.64		
Cycle: 19 0.5 µM				202.3	5.000E-7		30.00	1.496E+18	42.22		
Cycle: 20 0.75 µM				209.6	7.500E-7		30.00	1.496E+18	36.96		
Cycle: 21 1 µM				224.6	1.000E-6		30.00	1.496E+18	28.31		

Parameters table

Curve	ka (1/Ms)	SE(ka)	kd (1/s)	SE(kd)	Rmax (RU)	SE(Rmax)	Conc (M)	tc	SE(tc)	f (ul/min)	RI (RU)	SE(RI)
	5.678E+4	3.6E+2	0.001542	1.0E-5				4.815E+17	1.5E+21			
Cycle: 17 0.1 µM					120.9	0.74	1E-07			30	25.9	0.29
Cycle: 18 0.25 µM					172.3	0.56	2.5E-07			30	43.6	0.32
Cycle: 19 0.5 µM					202.3	0.37	5E-07			30	42.2	0.40
Cycle: 20 0.75 µM					209.6	0.33	7.5E-07			30	37.0	0.42
Cycle: 21 1 µM					224.6	0.33	1E-06			30	28.3	0.42

Kinetics: ‘HuRMut_La1’, fit: ‘1. 1:1 Binding’

Curve: Fc=2-1 Ligand: N/A Sample: HuRMut_La1 Temp: 25°C

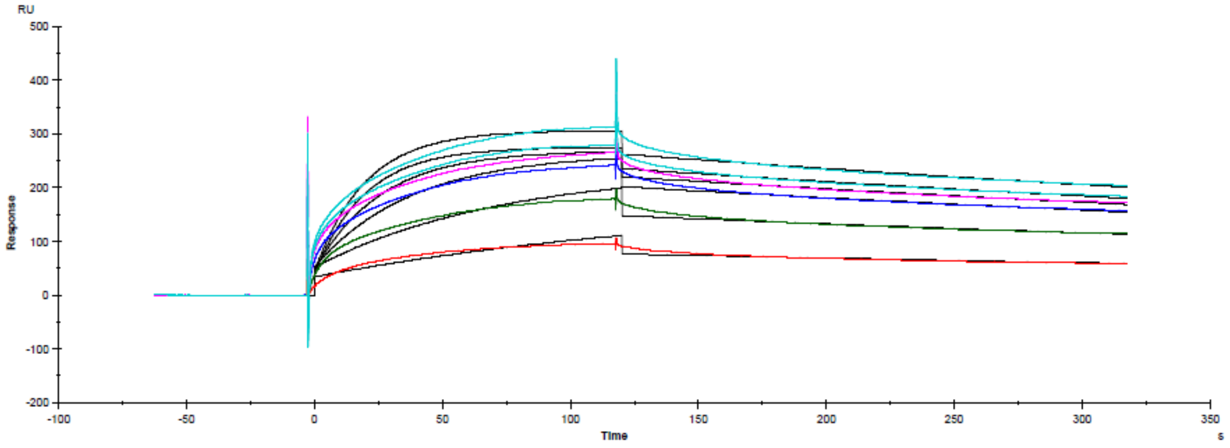

Residuals

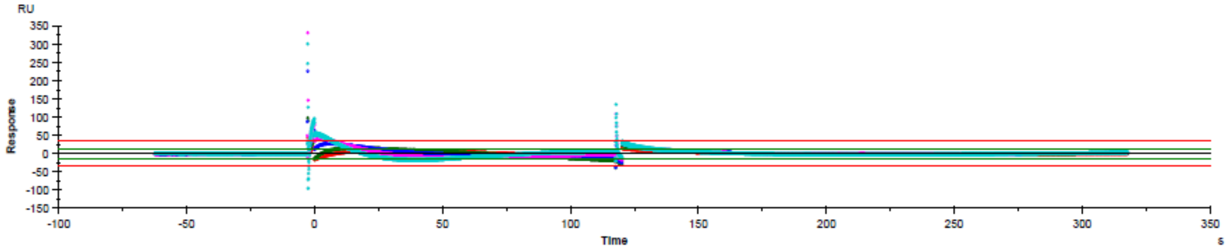

Kinetics: ‘HuRMut_La1’, fit: ‘1. 1:1 Binding’ (continued)

Report table

Curve	ka (1/Ms)	kd (1/s)	KD (M)	Rmax (RU)	Conc (M)	tc	Flow (ul/min)	kt (RU/Ms)	RI (RU)	Chi² (RU²)	U-value
	4.952E+4	0.001349	2.723E-8			8.528E+20				104	4
Cycle: 17 0.1 µM				184.4	1.000E-7		30.00	2.650E+21	34.17		
Cycle: 18 0.25 µM				202.9	2.500E-7		30.00	2.650E+21	51.73		
Cycle: 19 0.5 µM				222.5	5.000E-7		30.00	2.650E+21	52.86		
Cycle: 20 0.75 µM				230.6	7.500E-7		30.00	2.650E+21	46.52		
Cycle: 21 1 µM				242.3	1.000E-6		30.00	2.650E+21	39.16		
Cycle: 22 1 µM				270.1	1.000E-6		30.00	2.650E+21	43.24		

Parameters table

Curve	ka (1/Ms)	SE(ka)	kd (1/s)	SE(kd)	Rmax (RU)	SE(Rmax)	Conc (M)	tc	SE(tc)	f (ul/min)	RI (RU)	SE(RI)
	4.952E+4	2.8E+2	0.001349	8.9E-6				8.528E+20	9.0E+24			
Cycle: 17 0.1 µM					184.4	0.98	1E-07			30	34.2	0.33
Cycle: 18 0.25 µM					202.9	0.64	2.5E-07			30	51.7	0.36
Cycle: 19 0.5 µM					222.5	0.42	5E-07			30	52.9	0.42
Cycle: 20 0.75 µM					230.6	0.36	7.5E-07			30	46.5	0.45
Cycle: 21 1 µM					242.3	0.35	1E-06			30	39.2	0.45
Cycle: 22 1 µM					270.1	0.37	1E-06			30	43.2	0.47

Kinetics: 'La 2', fit: '1. 1:1 Binding'

Curve: Fc=2-1 Ligand: Biotin miR125 primary RNA Sample: La Temp: 25°C

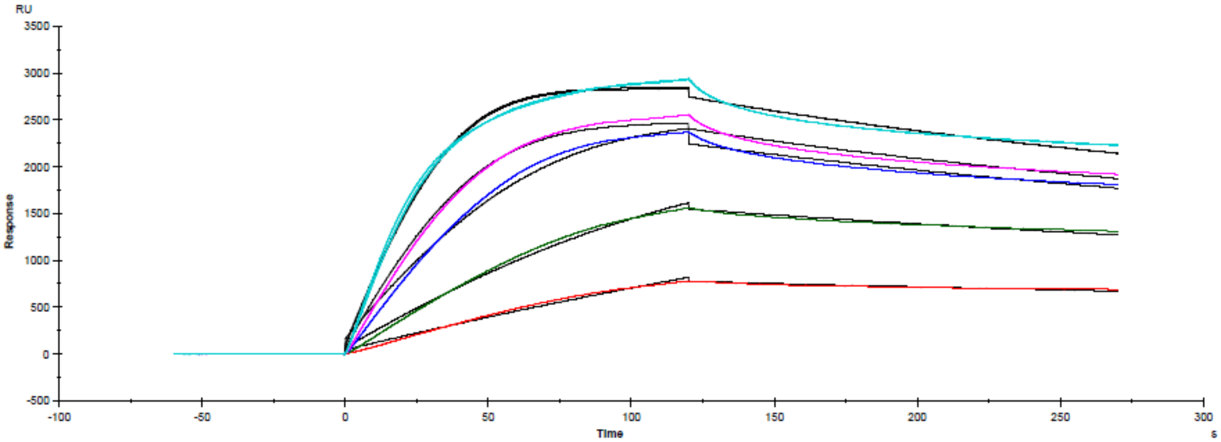

Residuals

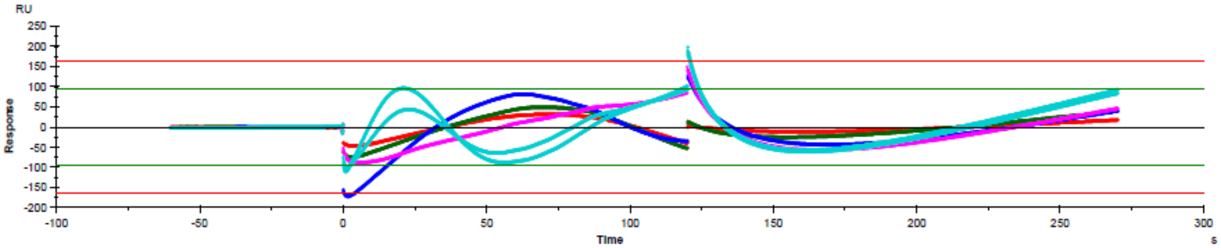

Kinetics: 'La 2', fit: '1. 1:1 Binding' (continued)

Report table

Curve	ka (1/Ms)	kd (1/s)	KD (M)	Rmax (RU)	Conc (M)	tc	Flow (ul/min)	kt (RU/Ms)	RI (RU)	Chi² (RU²)	U-value
	7.819E+5	0.002081	2.661E-9			3.805E+8				1.64E+3	2
Cycle: 7 0.01 µM				2476	1.000E-8		30.00	1.182E+9	42.99		
Cycle: 8 0.025 µM				2291	2.500E-8		30.00	1.182E+9	66.10		
Cycle: 9 0.05 µM				2464	5.000E-8		30.00	1.182E+9	162.5		
Cycle: 10 0.075 µM				2501	7.500E-8		30.00	1.182E+9	59.79		
Cycle: 11 0.1 µM				2820	1.000E-7		30.00	1.182E+9	84.12		
Cycle: 12 0.1 µM				2818	1.000E-7		30.00	1.182E+9	101.0		

Parameters table

Curve	ka (1/Ms)	SE(ka)	kd (1/s)	SE(kd)	Rmax (RU)	SE(Rmax)	Conc (M)	tc	SE(tc)	f (ul/min)	RI (RU)	SE(RI)
	7.819E+5	5.9E+3	0.002081	7.9E-6				3.805E+8	2.5E+6			
Cycle: 7 0.01 µM					2476.2	8.7	1E-08			30	43.0	1.3
Cycle: 8 0.025 µM					2290.9	4.9	2.5E-08			30	66.1	1.4
Cycle: 9 0.05 µM					2464.3	2.8	5E-08			30	162.5	1.6
Cycle: 10 0.075 µM					2500.9	1.8	7.5E-08			30	59.8	1.8
Cycle: 11 0.1 µM					2820.4	1.8	1E-07			30	84.1	1.8
Cycle: 12 0.1 µM					2817.8	1.8	1E-07			30	101.0	1.8
